# Atherosclerotic Risk Factor Prevalence in Adults With Congenital Heart Disease: A Meta-Analysis

**DOI:** 10.1016/j.jacadv.2024.101359

**Published:** 2024-10-19

**Authors:** Andreas S. Papazoglou, Konstantinos G. Kyriakoulis, Konstantinos Barmpagiannos, Dimitrios V. Moysidis, Anastasios Kartas, Maria Chatzi, Amalia Baroutidou, Vasileios Kamperidis, Antonios Ziakas, Konstantinos Dimopoulos, George Giannakoulas

**Affiliations:** a Athens Naval Hospital, Athens, Greece; b National and Kapodistrian University of Athens, School of Medicine, Athens, Greece; c First Department of Cardiology, General University Hospital of Thessaloniki AHEPA, Aristotle University of Thessaloniki, Greece; d 424 General Military Hospital of Thessaloniki, Thessaloniki, Greece; e Adult Congenital Heart Centre and Centre for Pulmonary Hypertension, Royal Brompton Hospital, Royal Brompton and Harefield Hospitals, Guy's and St Thomas' NHS Foundation Trust, London, United Kingdom; f National Heart and Lung Institute, Imperial College London, London, United Kingdom

**Keywords:** adults, atherosclerosis, congenital heart disease, cardiovascular prevention, prevalence, traditional risk factors

## Abstract

**Background:**

The risk of atherosclerotic cardiovascular disease (ASCVD) in adults with congenital heart disease (ACHD) is comparable to that of the general population and is driven by traditional ASCVD risk factors.

**Objectives:**

The aim of the study was to estimate the prevalence of traditional ASCVD risk factors (hypertension, dyslipidemia, diabetes mellitus [DM], obesity, smoking, and physical inactivity) in ACHD and compare it with the general population.

**Methods:**

A systematic literature search was conducted up to May 15, 2024, to identify studies (with or without control group) reporting the prevalence of ASCVD risk factors in ACHD. Meta-analyses were conducted to synthesize the prevalence of risk factors and compare it with that of the general population, where applicable.

**Results:**

We identified 62 studies (30 controlled) encompassing 110,469 ACHD (mean age 39 years; 52% males, 88% with simple/moderate congenital heart disease complexity). Of these, 54% (45%-63%) reported lack of regular exercise, 33% (26%-40%) had hypertension, 18% (14%-22%) were obese, 17% (11%-25%) had dyslipidemia, 12% (9%-14%) were current smokers, and 7% (5%-9%) had DM. The prevalence of ASCVD risk factors was similar in ACHD and controls, with the exception of DM (higher prevalence in ACHD) and smoking (lower prevalence in ACHD). Significant heterogeneity was observed among the included studies, partially explained by differences in age, congenital heart disease complexity, and the presence of cyanosis.

**Conclusions:**

Except for DM and smoking, the prevalence of traditional ASCVD risk factors is similar in ACHD compared to the general population. Further research is needed to determine whether interventions applied in the general population are also effective in ACHD.

Nearly 1 in 100 children is born with congenital heart disease (CHD).^1^ Advancements in medical care have allowed most of these patients to grow into adulthood, reaching ages where atherosclerosis becomes clinically relevant.^2^ Later in life, adults with congenital heart disease (ACHD) face an elevated risk of atherosclerotic cardiovascular disease (ASCVD) events, including myocardial infarction, stroke, chronic coronary artery disease, and ischemic heart failure.^3^

The risk of ASCVD in ACHD may even exceed that of the general population.^3^ It remains unclear whether this increased risk occurs because of an adverse risk factor profile in ACHD or in relation to the underlying congenital defect.^4^ Despite varying estimates concerning the prevalence of ASCVD risk factors in ACHD, the available data consistently underscore the independent impact of these risk factors on the clinical course of ACHD.^5^, ^6^, ^7^ The existing clinical consensus statements and guidelines for the management of ACHD in both Europe and the United States stress the importance of systematically addressing modifiable ASCVD risk factors in ACHD.^7^, ^8^, ^9^

Our systematic review aims to comprehensively evaluate the prevalence of traditional ASCVD risk factors in ACHD, based on the available literature. Additionally, we seek to compare the prevalence of ASCVD risk factors between the ACHD and the general population.

## Methods

### Search strategy and data sources

Our systematic review and meta-analysis was registered on the PROSPERO registry (CRD42023490437), and is reported according to the PRISMA 2020 statement (Supplemental Table 1).^10^

A systematic literature search was performed in PubMed, Scopus and Web of Science databases until May 15, 2024, using the algorithm “([ACHD] OR [congenital heart disease]) AND ([obesity] OR [hypertension] OR [dyslipidemia] OR [smoking] OR [physical activity] OR [diabetes]).” Reference lists of the articles retrieved and previous reviews were hand-searched.

### Study selection: eligibility criteria

Two trained reviewers (A.S.P. and K.B.) independently screened the titles, abstracts, and full articles for selection of studies aiming to investigate ASCVD risk factors in ACHD. Discrepancies were resolved by consensus with a third author (K.G.K.).

Studies were excluded if they were: 1) case reports or case series with <10 patients, reviews, editorials, conference abstracts, and practice guidelines; 2) conducted entirely in pediatric populations or did not provide data for the enrolled adult participants; and 3) published in non-English language. Studies including patients with established ASCVD (eg, prior myocardial infarction) were excluded from the meta-analysis of risk factor prevalence to avoid prevalence overestimation. In case of overlapping populations, the largest study providing the data of interest was included. Any study design (whether retrospective or prospective) and studies from all settings were considered, including those that were population-based, hospital-based, or from a CHD registry.

### Data extraction

The following data were independently extracted by 2 investigators (A.S.P. and K.B.), using prespecified forms: study design/population, demographic characteristics (age, sex, body mass index [BMI], CHD types/complexity), clinical/laboratory measurements (blood pressure, fasting glucose, glycated hemoglobin, carotid intima media thickness [CIMT], total cholesterol, low-density lipoprotein [LDL] cholesterol, high-density lipoprotein [HDL] cholesterol, and triglyceride levels), ASCVD risk factor (hypertension, diabetes mellitus [DM], dyslipidemia, obesity, smoking, lack of regular exercise) prevalence, and matching variables, where available. Continuous variables reported as median (IQR) were transformed to mean ± SD using appropriate formulas.^11^

### Quality assessment

The methodological quality of the included studies was independently assessed by 2 investigators (M.C. and A.B.) using relevant quality assessment tools for prevalence studies.^12^ The quality of evidence was assessed using the Grading of Recommendations Assessment Development and Evaluation (GRADE) tool.^13^

### Outcomes of interest

The primary outcome was the prevalence of ASCVD risk factors among ACHD. A secondary outcome was the comparison of risk factor prevalence between ACHD and general population controls.

### Statistical analysis

The data synthesis included the calculation of:

• Pooled proportions and 95% CIs of the ASCVD risk factors of interest (hypertension, DM, dyslipidemia, obesity, smoking, and lack of regular exercise) in ACHD populations;
• Pooled risk ratios (RRs) and 95% CIs using the DerSimonian and Laird method for the comparison of ASCVD risk factor prevalence among ACHD vs controls; and
• Mean differences and 95% CIs of continuous variables of interest (blood pressure, fasting glucose, glycated hemoglobin, CIMT, total/LDL/HDL cholesterol, and triglyceride levels) in ACHD vs controls.

A meta-analysis was carried out if ≥3 studies reported the outcome of interest. A random-effects model was a priori selected given the expected heterogeneity in study design and patients’ characteristics. Forest plots with proportions, RRs, and mean differences with corresponding 95% CIs are provided.

Heterogeneity was assessed using the Higgins and Thompson I^2^ and was quantified as low (<25%), moderate (25%-75%), or high (>75%).^14^ Potential publication bias was evaluated through the Egger’s test, the visual inspection of the relevant funnel plots, and trim-and-fill analyses.^15^

Meta-regression analyses were performed to estimate associations between the primary and secondary outcomes (response variables: ASCVD risk factor prevalence as proportions [%] and the comparison between ACHD and controls using RRs, respectively) and estimates of relevant covariates aggregated across different studies. These covariates included year of publication, sample size, mean age, male percentage, cyanotic percentage, and CHD complexity, provided they were reported in at least 10 studies. The model utilized an identity link function and assumed a normal distribution for the response variables. The model parameters included the regression coefficients for both the intercept and the covariates. To account for the multiple comparisons across the 6 meta-regression analyses performed per risk factor, a Bonferroni correction was applied to adjust the significance level, reducing it to α = 0.05/6 ≈ 0.00833. In this respect, the 95% CIs for the regression coefficients were adjusted accordingly. This adjustment was made to account for the inflated probability of type-1 error and to maintain robustness and validity in the presence of multiple statistical tests. Meta-regression analyses were performed to estimate associations between the primary and secondary outcomes (response variables: ASCVD risk factor prevalence as proportions [%] and the comparison between ACHD and controls using RRs, respectively) and estimates of relevant covariates aggregated across different studies. These covariates included year of publication, sample size, mean age, male percentage, cyanotic percentage, and CHD complexity, provided they were reported in at least 10 studies. The model utilized an identity link function and assumed a normal distribution for the response variables. The model parameters included the regression coefficients for both the intercept and the covariates. To account for the multiple comparisons across the 6 meta-regression analyses, a Bonferroni correction was applied to adjust the significance level, reducing it to α_adj_ = 0.05/6 ≈ 0.00833. Consequently, the 95% CIs for the regression coefficients were adjusted using this corrected significance level, ensuring that the adjusted CIs reflected the increased stringency. This adjustment was made to maintain robustness and validity in the presence of multiple statistical tests. Subgroup and sensitivity analyses were also performed: 1) in 100% cyanotic populations; 2) in 100% non-cyanotic populations; 3) in 100% coarctation of aorta (CoA) populations; 4) in age- and gender-matched controlled studies; and 5) after excluding cohort studies with extremely large populations (>10,000 study participants) to account for the potential sample size bias.

Statistical analyses were performed using Review Manager (RevMan, The Cochrane Collaboration, 2020) Version 5.4 and the “meta” and “metafor” packages of the R 4.2.2 software. A 2-tailed *P* value <0.05 was deemed as the statistical significance level.

## Results

### Search results and study selection

**Figure 1:**
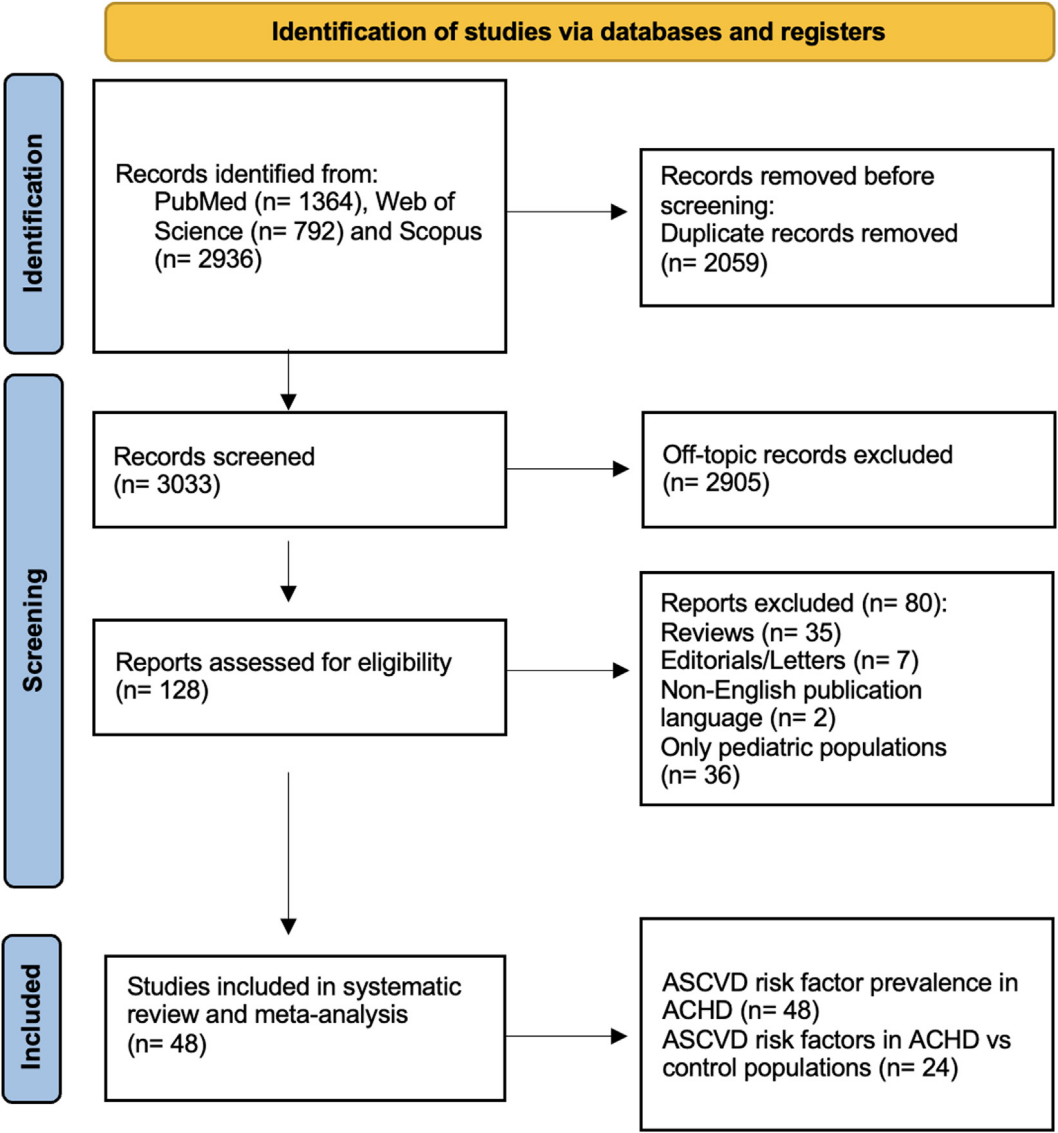
**PRISMA Flowchart of the Systematic Review Process** PRISMA 2020 flow diagram. ACHD = adults with congenital heart disease; ASCVD = atherosclerotic cardiovascular disease; PRISMA = Preferred Reporting Items for Systematic reviews and Meta-Analyses.

Study selection is depicted in Figure 1. Of the 13,232 studies initially identified, 62 full-text articles were deemed eligible for inclusion in this systematic review and meta-analysis (all provided data for the meta-analysis of ASCVD risk factor prevalence and 30 provided data for the comparison of ASCVD risk factors between ACHD and general population controls).

### Characteristics of the included studies

The main characteristics of the 62 included studies are summarized in Tables 1 and 2 according to the presence or not of a control group. The definitions used for ASCVD risk factor categorization in each of the included studies are detailed in the Supplemental Table 2. Most studies utilized prior diagnoses and International Classification of Diseases codes or treating medications for hypertension, DM, dyslipidemia coding, BMI values for obesity coding, and self-reported questionnaires for smoking and physical inactivity coding.

**Table 1:** Eligible Studies Reporting ASCVD Risk Factor Prevalence While Having Both ACHD Group and Control Group From the General Population

First Author, Year	Country	N	Males (%)	Age (y)	Complexity of CHD				Risk Factors Reported (ACHD vs Controls)					
					S	M	G	U	HTN	DM	DLD	Obesity	Smoking	Lack of Exercise
Moons, 2006	Belgium	1,976/1,071	54/NR	26/NR					**↓**	**↔**		**↑**	**↓**	**↓**
Vriend, 2006	the Netherlands	137/46	65/46	32/31	100	0	0	0	**↑**				**↑**	
Martínez-Quintana, 2010	Spain	158/152	60/32	28/33	8	72	20	0		**↔**				
Duffels, 2010	the Netherlands	54/54	56/54	38/37	0	0	100	0	**↓**				**↓**	
Zomer, 2012	the Netherlands	1,496/6,810	52/45	39/35	46	44	10	0		**↑**		**↓**	**↓**	**↔**
Ohuchi, 2014^b^	Japan	444/27	36/44	26/27	0	58	42	0						
Dellborg, 2015	Sweden	833/4,165	64/NR	70/70	0	100	0	0	**↔**		**↔**		**↓**	**↔**
Moon, 2015	South Korea	135/135	44/44	47/47	67		33	0	**↔**	**↔**	**↔**	**↔**	**↓**	**↑**
Sandberg, 2015	Sweden	2,424/4,605	58/49	31/NR	78		22	0				**↑**		
Madsen, 2016	USA	5,149/49,968	47/47	NR/NR	38	23	21	18		**↑**				
Caruana, 2016	Malta	125/375	52/52	31/31	15	68	17	0					**↓**	**↑**
Deen, 2016	USA	448/448	49/49	32/32	19		81	0	**↓**	**↑**	**↑**	**↔**		
Fedchenko, 2017	Sweden	278/183	NR/NR	NR/NR	83		17	0	**↓**	**↓**				
Trojnarska, 2017^b^	Poland	36/35	47/45	42/39	0		0	100						
Lerman, 2017	USA	1,451/1,451	50/50	52/52	69		21	0				**↔**		
Flannery, 2018	USA	248/744	52/52	51/51	0	88	12	0	**↑**	**↔**	**↔**	**↓**	**↓**	
Pickard, 2018	USA	174/5,472,242	80/60	60/68	0	100	0	0	**↔**	**↔**	**↔**		**↑**	
Tarp, 2018	Denmark	74/74	43/43	50/50	0	0	100	0		**↔**	**↔**		**↔**	**↑**
Saha, 2019	United Kingdom	2,006/497,983	59/46	58/58	100		0	0	**↑**	**↑**	**↑**	**↑**	**↔**	**↑**
Martínez- Quintana, 2019	Spain	818/1955	56/53	33/30	56	28	16	0	**↑**	**↑**		**↓**	**↓**	
Krishnamurthy, 2019	Germany	131/259	54/55	46/47	0	100	0	0	**↑**	**↔**		**↓**	**↓**	
Tarp, 2019^a^	Denmark	15/14	40/56	53/53	0	0	100	0	**↓**					
Zaqout, 2019	Belgium	539/1737	54/48	32/39	34	52	13	0	**↓**	**↓**	**↓**	**↔**	**↓**	
Larsson, 2019	Sweden	75/42	61/62	38/37	52	0	48	0					**↔**	**↔**
Malavazos, 2019	Italy	1,388/145,992	44/51	41/NR	60	31	9	0				**↔**		
Gales, 2020	USA	297/297	44/44	44/44	50	32	18	0	**↔**	**↓**	**↓**		**↔**	
Fox, 2021	USA	744/14,350	41/NR	34/NR	18	48	34	0					**↓**	
Lubert, 2021^b^	USA	164/81	58/25	29/34	0	0	100	0						
Umapathi, 2022	USA	6,720/7,359,470	39/42	NR/NR	0	74	26	0	**↑**	**↑**	**↑**	**↑**	**↓**	
Bjork, 2024	Sweden	24,699/270,961	51/52	NR/NR						**↑**				
Total (pooled)	Europe: 19/30, 63% USA: 9/30, 30% Asia: 2/30, 7%	53,296/13,837,263	50/50	38/34	77		21	2	**↔**	**↑**	**↔**	**↔**	**↓**	**↔**

ACHD = adults with congenital heart disease; CHD = congenital heart disease; DLD = dyslipidemia; DM = diabetes mellitus; G = great; HTN = hypertension; M = moderate; NR = not reported; S = simple; U = unclassified.

Data are presented as ACHD/controls. Blank cells indicate that the variable of interest is not reported. Arrows indicate whether the percentage of a risk factor is statistically significantly increased or decreased in ACHD vs controls.

a The study by Tarp et al (2019) was included in Table 1 despite potential overlap with the study by Tarp et al (2018), because the former provided additional data not reported in the initial study (eg, hypertension rates in ACHD vs controls).

b The studies by Trojnarska et al, Ohuchi et al, and Lubert et al did not provide rates of ASCVD risk factors in ACHD vs control groups; however, they were included in the systematic review and meta-analysis because they studied ASCVD burden in ACHD vs controls and provided continuous measurements of the interest, including body mass index, serum lipidemic values, and/or carotid intima media thickness.

**Table 2:** Eligible Studies Reporting ASCVD Risk Factor Prevalence in ACHD Without Having a Control Group From the General Population

First Author, Year	Country	N	Males (%)	Age (y)	Complexity of CHD				Risk Factors Reported					
					S	M	G	U	HTN	DM	DLD	Obesity	Smoking	Lack of Exercise
Dua, 2007	UK	61	59	32	85		15	0				✓		✓
Engelfriet, 2007	the Netherlands	3,375	NR	28									✓	
Giannakoulas, 2009	UK	250	53	51	59	29	12	0	✓	✓	✓		✓	
Zaidi, 2011	USA	165	53	31	32		68	0	✓			✓		
Roifman, 2012	Canada	7,237	31	45	100		0	0	✓	✓	✓			
Buys, 2013	Belgium	103	68	29	0	100	0	0				✓	✓	✓
Sandberg, 2013	Sweden	315	71	33	0	100	0	0					✓	✓
Luijendijk, 2014	the Netherlands	160	64	32	0	100	0	0	✓	✓	✓			
Karsenty, 2015	France	135	39	40	45	37	18	0					✓	✓
Lanz, 2015	Canada	12,440	45	50	90		10		✓	✓	✓	✓	✓	
Chung, 2016	USA	129	55	28	0	0	100	0				✓		
Brida, 2017	UK	3,069	52	32	33	44	23	0				✓		
Muller, 2017	Germany	786	57	31	13	31	51	5						✓
Lui, 2018	USA	178	51	37	0	57	43	0	✓	✓	✓	✓	✓	✓
Hacker, 2018	Germany	551	52	44	14	28	58	0	✓	✓			✓	✓
Harris, 2018	Canada	102	54	35	30	47	23	0	✓	✓		✓	✓	✓
Bokma, 2018	the Netherlands	333	63	55	54	34	12	0	✓	✓	✓	✓	✓	✓
Bauer, 2018	Germany	539	51	38	34	35	29	2	✓	✓	✓	✓	✓	
Egbe, 2019	USA	105	61	47	0	100	0	0	✓	✓	✓		✓	
Johnson, 2019	USA	73	53	44	100		0	0	✓	✓	✓		✓	
Egbe, 2019 (2)	USA	1,530	50	37	0	100	0	0	✓	✓	✓		✓	
Jackson, 2020	USA	1863	NR	NR								✓		
Holbein, 2020	Multicenter	4,028	47	33	26	49	25	0					✓	✓
Murakami, 2021	Japan	64	53	NR	0	78	9	13	✓				✓	
Meijs, 2021	the Netherlands	920	60	24	0	100	0	0	✓	✓	✓		✓	
Kwiatek-Wrzosek, 2021	Poland	322	34	66	82	14	4	0	✓	✓	✓		✓	
Misra, 2022	USA	261	68	28	23	43	34	0				✓		
Jepson, 2022	USA	1,070	51	31	18	51	31	0	✓			✓	✓	
Egbe, 2022	USA	5,025	51	35	6	62	26	6	✓	✓	✓	✓	✓	
Levene, 2023	USA	13,896	48	NR	74	26		0	✓	✓	✓	✓	✓	
Garcia Cruz, 2023	Mexico	1,171	64	32	45	38	17	0	✓	✓	✓	✓	✓	✓
Kowalik, 2024	Poland	144	35	66	74	20	6	0	✓	✓	✓		✓	
Total (pooled)^a^	North/SouthAmerica: 15/32, 47% Europe: 15/32, 47% Asia: 1/32, 3% Multicenter: 1/32, 3%	110,469	52	39	88		11	1	0.33 (0.26, 0.40)	0.07 (0.05, 0.09)	0.17 (0.11, 0.25)	0.18 (0.14, 0.22)	0.12 (0.09, 0.14)	0.54 (0.45, 0.63)

ACHD = adults with congenital heart disease; CHD = congenital heart disease; DLD = dyslipidemia; DM = diabetes mellitus; Exer = exercise; G = great; HTN = hypertension; M = moderate; NR = not reported; S = simple; Smok = smoking; U = unclassified.

Check marks indicate whether the prevalence of a risk factor is reported in a study.

Blank cells indicate that the variable of interest is not reported.

a The total (pooled) values and percentages have been calculated taking also into consideration the ACHD populations of the controlled studies in Table 1 (total: n = 62 studies).

Regarding controlled studies (n = 53,296/13,837,263, mean age 38/34 years, males 50%/50% for ACHD/controls, respectively, and simple-moderate/great/unclassified CHD complexity for ACHD 77%/22%/2%, respectively),^16^, ^17^, ^18^, ^19^, ^20^, ^21^, ^22^, ^23^, ^24^, ^25^, ^26^, ^27^, ^28^, ^29^, ^30^, ^31^, ^32^, ^33^, ^34^, ^35^, ^36^, ^37^, ^38^, ^39^, ^40^, ^41^, ^42^, ^43^, ^44^, ^45^ most were conducted after 2010 in Europe, while one-third was conducted in the United States (Table 1). Regarding uncontrolled studies (n = 110,469, mean age 39 years, males 52%, and simple-moderate/great/unclassified CHD complexity 88%/11/1%, respectively, including the ACHD of the studies with control groups),^46^, ^47^, ^48^, ^49^, ^50^, ^51^, ^52^, ^53^, ^54^, ^55^, ^56^, ^57^, ^58^, ^59^, ^60^, ^61^, ^62^, ^63^, ^64^, ^65^, ^66^, ^67^, ^68^, ^69^, ^70^, ^71^, ^72^, ^73^, ^74^, ^75^, ^76^, ^77^ most of them were also conducted after 2010, originating equally from Europe and North/South America (Table 2).

### Quality assessment and risk of bias

The risk of bias was considered to be low in most of the included studies (Supplemental Table 3).

### Primary outcome: ASCVD risk factor prevalence

**Figure 2:**
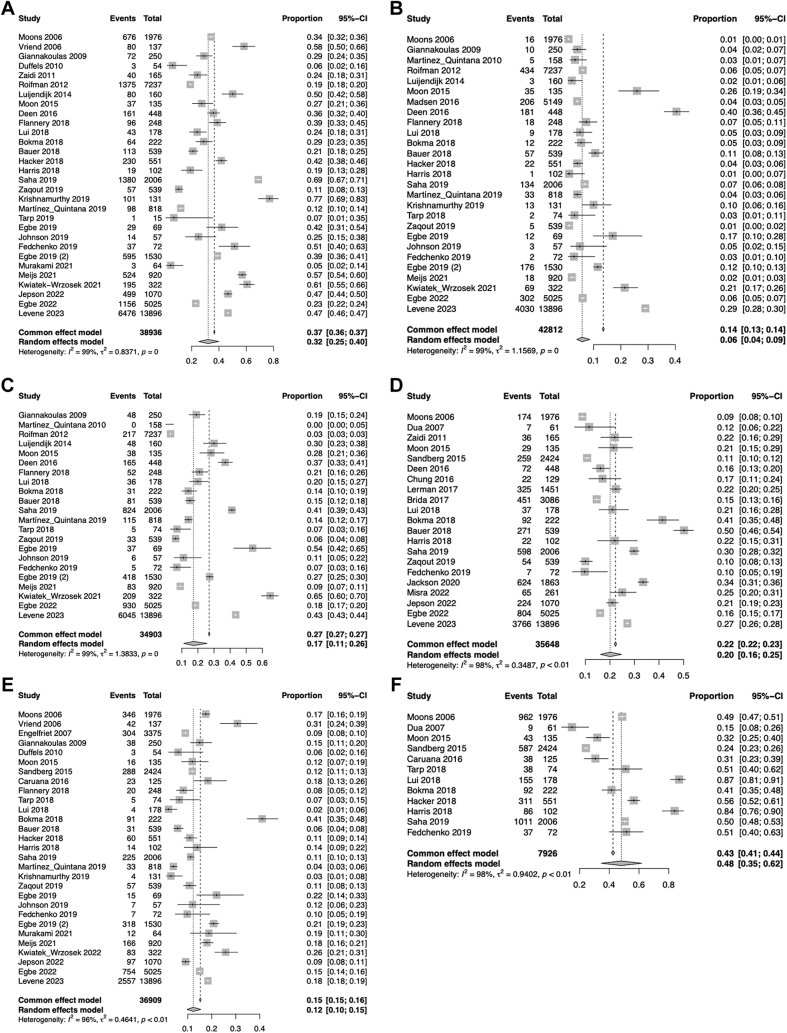
**Meta-Analysis of Proportions for the Primary Study Outcome: Prevalence of Atherosclerotic Risk Factors in Adults With Congenital Heart Disease** Pooled prevalence of atherosclerotic risk factors in adults with congenital heart disease. (A) Hypertension, (B) diabetes mellitus, (C) dyslipidemia, (D) obesity, (E) smoking, (F) lack of regular exercise.

Meta-analysis of the 62 eligible studies (Tables 1 and 2) showed that almost 1 in 3 (33%) ACHD had hypertension; 7% had DM; 1 in 6 (17%) had dyslipidemia; 1 in 5 (18%) were obese; and 1 in 8 (12%) were current smokers. Additionally, almost one-half (54%) of the studied ACHD reported no regular physical activity. In each of these analyses, a high degree of heterogeneity was observed (I^2^ >75%). The demographic characteristics of the studied population as well as the outcomes of the meta-analysis are presented in Figure 2 and Supplemental Table 4.

### Sensitivity analyses

#### Subgroup analyses

ASCVD risk factor prevalence was also examined in 3 subgroups: 1) 100% cyanotic populations (5 studies, n = 338); 2) 100% noncyanotic populations (14 studies, n = 13,112); and 3) 100% patients with CoA (8 studies, n = 2,933) (Supplemental Table 4). Hypertension was more prevalent in populations with CoA (57% [49%-64%]) compared to noncyanotic (47% [37%-58%]) and cyanotic populations (5% [2%-10%]). Dyslipidemia (20% [11%-35%]) was more prevalent in noncyanotic populations compared to the overall ACHD population (17% [11%-25%]) or populations with CoA (14% [7%-25%]). Finally, smoking was less frequently reported in cyanotic (5% [2%-10%]) than in noncyanotic (11% [7%-17%]) populations.

#### Meta-regression analyses

In the meta-regression analyses, the following associations were observed (Supplemental Table 5).

a. A 1-year increase in the mean population age was associated with a 0.8% relative increase in hypertension prevalence (Supplemental Figure 1) and a 1.1% relative increase in dyslipidemia prevalence (Supplemental Figure 2).
b. A 1% increase in the female population percentage was linked with a 1% relative increase in dyslipidemia prevalence (Supplemental Figure 3).
c. Α 1% increase in the cyanotic population percentage was associated with a 0.4% relative decrease in hypertension prevalence (Supplemental Figure 4).
d. A 1% increase in the proportion of patients with great complexity CHD was associated with a 0.2% relative decrease in smoking prevalence (Supplemental Figure 5).

#### Publication bias assessment

Egger’s test did not suggest the existence of potential publication bias (all *P* > 0.05); however, visual inspection of the funnel plots indicated the potential presence of publication bias (Supplemental Figure 6). The trim-and-fill method suggested that our prevalence meta-analyses might have been influenced by publication bias. The filled studies—despite their small number—often had negative or lower effect sizes compared to the original studies, indicating that the initial observed effects could have been inflated due to selective reporting or publication bias.

### Secondary outcome: comparison of ASCVD risk profile between ACHD and controls

**Figure 3:**
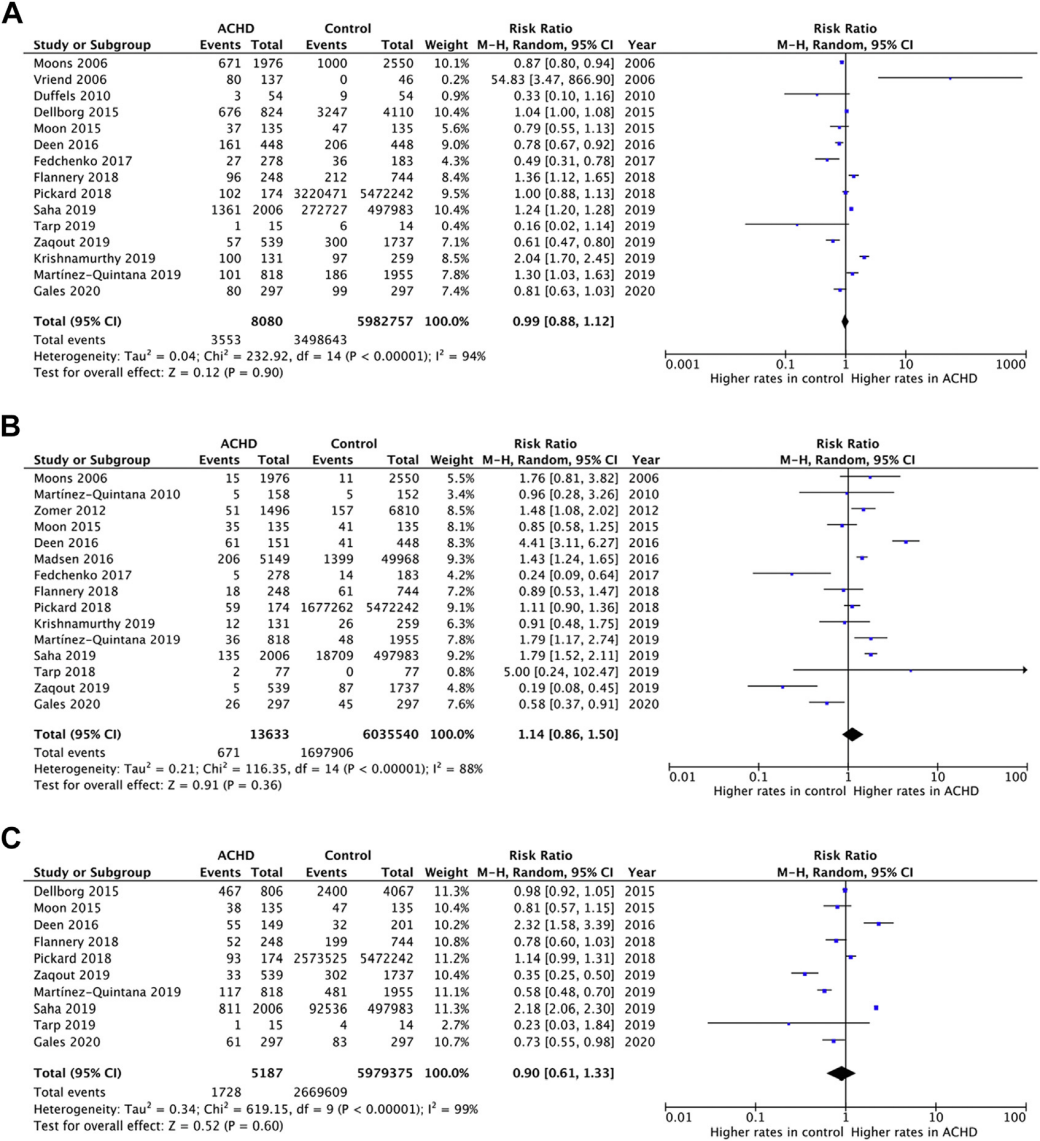

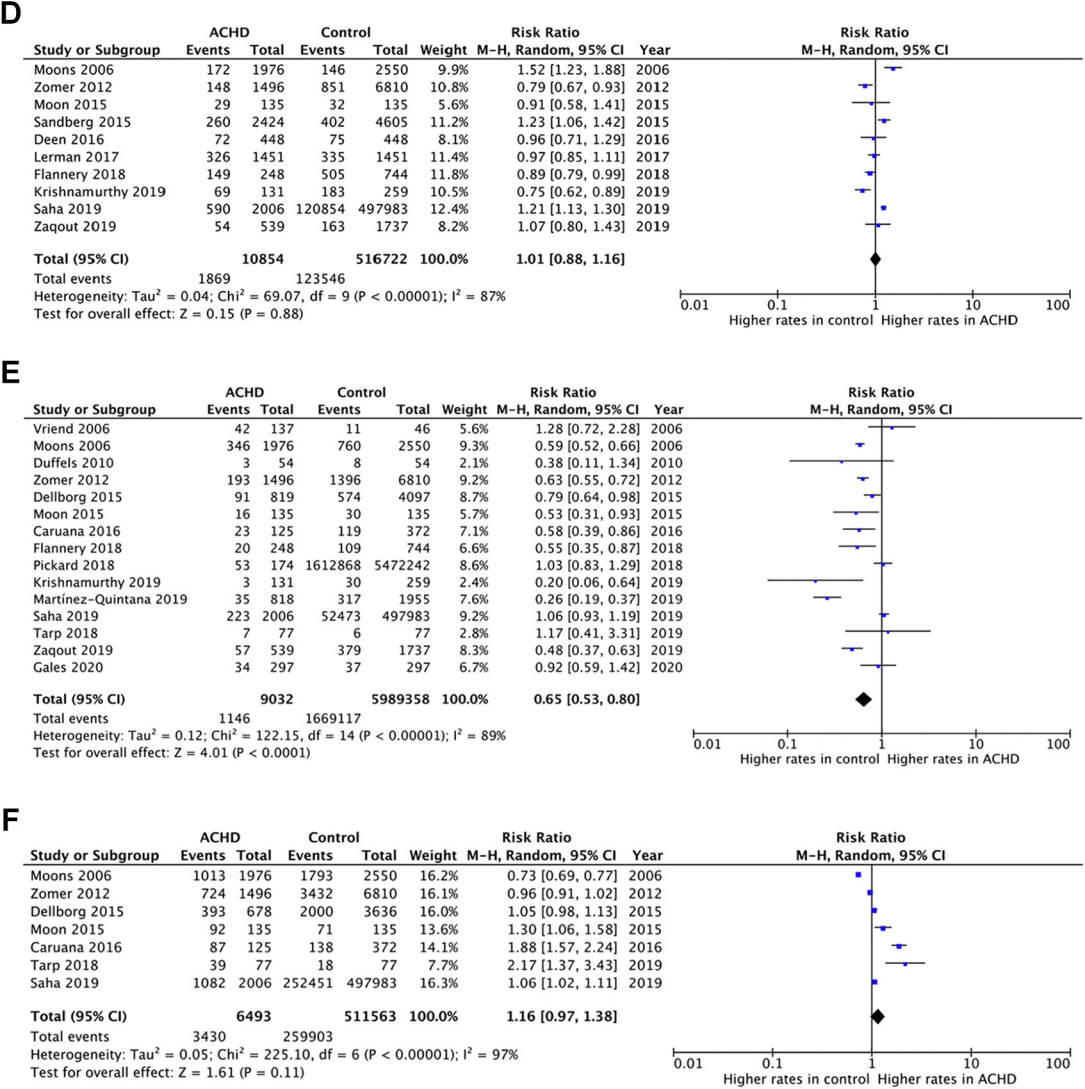
**Forest Plots of Risk Ratios for Atherosclerotic Risk Factor Prevalence in Adults With Congenital Heart Disease Compared to General Population Controls** Risk comparison for atherosclerotic risk factors between ACHD and controls. (A) Hypertension, (B) diabetes mellitus, (C) dyslipidemia, (D) obesity, (E) smoking, (F) lack of regular exercise. ACHD = adults with congenital heart disease.

The prevalence of ASCVD risk factors among ACHD (n = 53,296: 50% male, 8% cyanotic, 21% great CHD complexity) was compared to controls in 30 studies (n = 13,837,263: 50% male) (Table 1, Supplemental Table 6, Figure 3). We found no significant difference between the 2 populations in terms of hypertension (RR: 1.02 [95% CI: 0.92-1.12]), dyslipidemia (RR: 0.99 [95% CI: 0.76-1.27]), obesity (RR: 1.01 [95% CI: 0.92-1.12]), or lack of regular exercise (RR: 1.16 [95% CI: 0.98-1.37]); however, there was a higher prevalence of DM (RR: 1.30 [95% CI: 1.09-1.55]) and a lower frequency of smoking (RR: 0.67 [95% CI: 0.57-0.80]) in the ACHD vs controls.

In this setting, we also compared clinical variables extracted from both ACHD and general population controls (Supplemental Table 7). ACHD and controls had similar age, fasting blood glucose, glycated hemoglobin, serum triglycerides, CIMT, and blood pressure values. However, total cholesterol (−19.22 mg/dL [−25.70 to −12.75]), LDL (−9.62 mg/dL [−15.16 to −4.08]), HDL (−8.73 mg/dl [−12.30 to −5.15]) cholesterol levels, and BMI [−0.76 kg/m^2^ (−1.07 to −0.44)] were significantly lower in ACHD. The degree of heterogeneity in all the above analyses was considerable (I^2^ >75%).

### Sensitivity analyses

#### Subgroup analyses

In the subgroup analyses (Supplemental Table 6), the analysis of 100% cyanotic and 100% noncyanotic populations altered the main outcome in terms of the prevalence of hypertension: cyanotic ACHD had significantly lower prevalence of hypertension compared to controls in 3 studies (RR: 0.18 [95% CI: 0.06-0.53]). Conversely, noncyanotic ACHD had a significantly higher prevalence of hypertension compared to their controls in 5 studies (RR: 1.35 [95% CI: 1.04-1.74]). Moreover, 100% cyanotic populations had lower systolic blood pressure (−8.44 mm Hg [−15.71 to −1.16]) than their counterparts from the general population. Noncyanotic ACHD had increased diastolic blood pressure values (2.73 mm Hg [0.49-4.98]) compared to the control populations and did not differ in lipid values. After excluding the studies with a very large sample size (≥10,000), DM prevalence was found to be similar in ACHD vs controls (12 studies) (Supplemental Table 6).

#### Meta-regression analyses

Our meta-regression analyses for the risk of ASCVD risk factors (Supplemental Table 8) yielded that a 1% increase in the proportion of patients with great complexity CHD was associated with: 1) a 1% relative decrease in the risk of hypertension (Supplemental Figure 7); and 2) a 1.6% relative increase in the risk of DM (Supplemental Figure 8).

#### Publication bias assessment

Egger’s test did not suggest significant publication bias (all *P* > 0.05); however, trim-and-fill analyses were performed due to the visual inspection of the funnel plots (Supplemental Figure 9). The trim-and-fill analyses suggested varying degrees of publication bias. For dyslipidemia, the addition of only one imputed study points to a lack of significant publication bias. For smoking and obesity, the minimal impact of imputed studies on effect sizes indicates that publication bias is unlikely to have affected the results. For DM and hypertension, the analyses suggest possible publication bias, which did not alter the initial outcomes.

### GRADE appraisal of the quality of evidence

The quality of evidence provided as assessed with the GRADE tool (Supplemental Table 9) yielded outcomes of “very low” certainty, due to the observational nature of the included studies and the increased between-study heterogeneity.

## Discussion

This meta-analysis is the first to systematically evaluate the whole spectrum of ASCVD risk factors in ACHD and included 110,469 ACHD with mostly simple/moderate CHD complexity. In this young population, there was a high prevalence of hypertension, dyslipidemia, and obesity, with 7% having DM and 12% smoking. Over one-half were not exercising regularly. The risk of hypertension was particularly elevated in CoA and noncyanotic populations. Our secondary analyses demonstrated that except for DM, the remaining cardiovascular risk factors were not more prevalent in ACHD compared to the general population (Central Illustration).

**Central Illustration:**
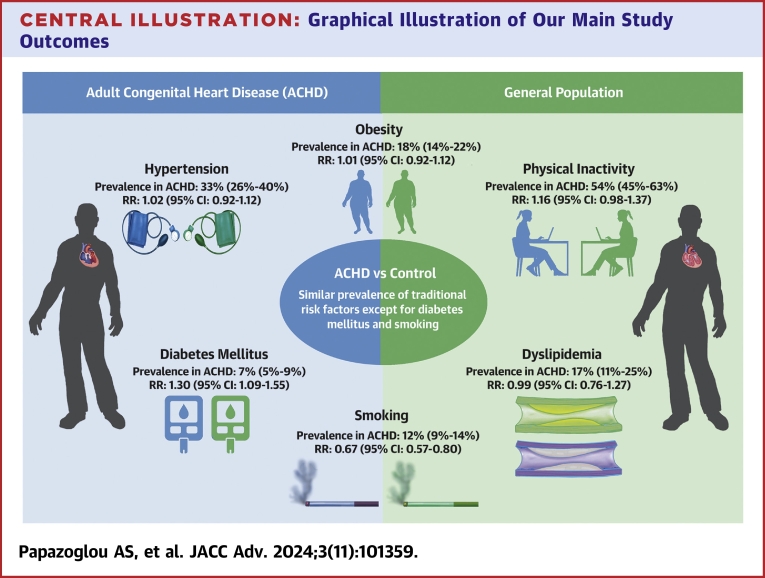
**Graphical Illustration of Our Main Study Outcomes** Atherosclerotic risk factors in adults with congenital heart disease. ACHD = adults with congenital heart disease; RR = risk ratio.

### Arterial hypertension

Hypertension prevalence is known to be age-dependent, and this was also demonstrated in our meta-regression analysis. The age-standardized prevalence of hypertension in the general European adult population is estimated to be 24% (29% in males and 19% in females),^78^ which concurs with our ACHD findings. In ACHD populations, determining the true hypertension prevalence may be challenging, since many ACHD are receiving long-term cardiac medications with antihypertensive properties. As expected, the highest prevalence of hypertension was observed in CoA patients, given that these patients have a high prevalence of residual hypertension even after successful repair of the underlying lesion.^48^ Patients with CoA were among the most commonly included individuals in the simple/moderate complexity noncyanotic subgroup and probably influenced the negative association between greater CHD complexity or cyanosis and hypertension.

The diagnostic and therapeutic approach to hypertension in ACHD is similar to that of non-ACHD populations, with emphasis on annual screening (office and out-of-office blood pressure measurements) and behavior modification.^79^ Regarding antihypertensive pharmacotherapy, the clinician should always consider the underlying CHD and any related issues (eg, caution using vasoactive medications in Eisenmenger syndrome).

### Diabetes mellitus

The prevalence of DM in the ACHD population was 7% (5%-9%) in our meta-analysis, higher than the corresponding prevalence in age-matched general population.^80^ DM is expected to become more prevalent as the ACHD population ages. A positive association between CHD of great complexity and the risk ratio for DM was observed, which underscores the observation by Ohuchi et al that patients with complex CHD might develop insulin resistance despite normal fasting glucose levels.^81^ It has been suggested to screen all ACHD (particularly those with complex CHD) at least once every 3 years for DM (or annually if there are evidence of “prediabetes”) measuring glycated hemoglobin or oral glucose tolerance test.^79^

### Obesity

One-fifth of the ACHD population was obese, which is similar to the general population at least in the United States.^82^ Previous authors have suggested that metabolic syndrome is more frequent in ACHD compared to age- and sex-matched controls (due to sedentary lifestyle along with self- or physician-imposed exercise restrictions^83^^,^^84^), but this was not confirmed in our study. Unfortunately, our analysis could not provide evidence for specific ACHD subgroups, such as patients with Fontan circulation or cyanotic CHD, who are less likely to become obese compared to other ACHD populations.^85^

Obesity is a major determinant of outcome in the ACHD population, with substantial health and economic implications.^86^ It also impacts perioperative outcomes and eligibility for cardiac transplantation.^87^ Relevant interventions and counseling should be promoted.

### Dyslipidemia

Dyslipidemia was observed nearly in one every 6 ACHD, despite the relatively young average age of the studied population. Interestingly, the subgroup analysis on noncyanotic populations showed higher rates of dyslipidemia compared to the overall ACHD population.^88^ However, the lower total cholesterol levels in cyanotic ACHD were due to lower HDL levels, while LDL levels were not significantly lower than in control patients. Hence, hypotheses of a protective effect of cyanosis against dyslipidemia were not supported by our results.^89^^,^^90^

Recent reports suggest that ACHD are less likely to receive statins for primary prevention than matched controls.^27^^,^^52^ Potential explanations for this “undertreatment” may be the “fear of polypharmacy” or the fact that ACHD are often followed by pediatric cardiologists who are less accustomed to managing cardiovascular risk factors in adults. Future studies are needed to understand whether the setting of ACHD care affects adherence to cardiology guidelines. A fasting lipid panel is recommended every 5 years to screen for dyslipidemia in all ACHD.

### Smoking

Tobacco smoking is a major modifiable risk factor advancing cardiovascular mortality by more than 5 years.^91^ Fortunately, the proportion of smoking ACHD seems to be smaller than in the general population, possibly due to the lifelong education they receive.^37^ Nevertheless, it is worrisome that there is still a sizeable proportion (9%-14%) of ACHD smokers that needs to be addressed. Education as part of the transition process should be introduced after the age of 12 years, and assistance for patients who do smoke should achieve the target of making smoking extinct in the ACHD population.^9^

### Physical inactivity

Lack of regular exercise was rather common in this meta-analysis, but not dissimilar to the general population. Since physical inactivity rates depend on self-reporting, our outcomes could potentially underestimate the actual physical inactivity rates in ACHD. Moreover, the assessment of physical activity differed among studies. Half of the included studies coded physical activity according to the European Society of Cardiology recommendations on physical activity (ie, 3-5 times per week of moderate intensity exercise). The rest utilized different criteria for regular physical activity characterization (eg, >1 hour per week or using the Physical Activity Questionnaire). Research on physical activity among ACHD is limited,^92^ and the available data point to a clear beneficial effect of exercise in most ACHD. According to a scientific statement from the American Heart Association in 2013,^92^ very few ACHD are sufficiently limited by residual hemodynamic or other abnormalities to impede physical activity or necessitate significant restrictions to exercise. The dangers of an overcautious approach to exercise are well-described.^93^

### ASCVD risk estimation

ACHD, and especially those with CHD of great complexity,^94^ have been found to be at increased risk of ASCVD^3^^,^^4^ and dismal prognosis.^95^ However, the prevalence of most ASCVD risk factors in ACHD appears to be similar to that of the general population. This suggests that CHD *per se,* at least for particular lesions, or the case of undertreatment might predispose patients to increased ASCVD risk.^4^ Early identification and optimal management of ASCVD risk factors are of utmost importance to minimize morbidity and mortality in this population. For this aim, robust ASCVD risk scores that apply to CHD patients are necessary, since the most frequently used risk scores are not derived and validated in ACHD. Future research will hopefully provide further insight into ACHD-centered risk stratification and prevention strategies.

### Strengths and limitations

The strength of this meta-analysis is the relatively large number of eligible cohort studies encompassing a total of 110,469 ACHD, which provide reliable, precise estimates on the prevalence of ASCVD risk factors. All included studies were published in the past 18 years (92% after 2010); hence, our findings reflect and are generalizable to current practice.

The limitations of our meta-analysis are primarily related to the observational nature of the eligible studies and the heterogeneity of ACHD populations. Given that this is a study-level meta-analysis, we were unable to study the prevalence of each ASCVD risk factor for specific CHD groups. We should not ignore the potential publication bias and the potential impact of study sample size on our meta-analytic outcomes. The exclusion of very large cohorts when performed as a sensitivity analysis altered the significance of the risk ratio for DM in ACHD, making us doubt for potential sample size bias. However, meta-regression and subgroup analyses at least in part addressed this limitation. Moreover, the between-study heterogeneity in key exposure variables (ie, varied CHD types, underlying physiologies, operative approaches, and postoperative sequelae) was marked, and, therefore, patient-level meta-analyses may best inform lesion-specific prevalence of ASCVD risk factors in ACHD.

Accounting for age and sex when defining ASCVD risk factors in ACHD is crucial.^25^ This was achieved only in a subgroup analysis since not every ACHD study used age- and sex-matched controls. Furthermore, although an attempt was made to calculate the prevalence of ASCVD risk factors in ACHD without prior established ASCVD, there may still be some patients with ASCVD at baseline in the included studies.

Our analyses rely on the prevalence of ASCVD risk factors based on the coding used by each investigator. However, this coding varied between studies (eg, different diagnostic cut-off values or criteria, International Classification of Diseases diagnoses, medication history). This inconsistency in variable coding among studies reduces the value of synthesized data and makes it more difficult to draw reliable conclusions about the prevalence of ASCVD risk factors in ACHD. Finally, reporting bias might exist in self-reported and not objectively collected variables, such as smoking and physical activity.

## Conclusions

DM prevalence was higher and smoking frequency was lower in ACHD compared to controls; the remaining traditional ASCVD risk factors were as prevalent as in the general population. As ACHD age, exposure to ASCVD risk will increase exponentially. Further research is needed to determine whether these risk factors are associated with adverse outcomes in ACHD and to assess the potential clinical benefits and cost-effectiveness of early DM screening and education on lifestyle modification in this population.

Perspectives **COMPETENCY IN MEDICAL KNOWLEDGE:** This meta-analysis is the first to systematically evaluate the prevalence of traditional cardiovascular risk factors in ACHD. Hypertension, dyslipidemia, obesity, and physical inactivity had similar rates in both ACHD and general population. Diabetes mellitus was more prevalent, and smoking was less prevalent among ACHD. The observed heterogeneity was partially explained by meta-regressions on the mean age and the percentage of great CHD complexity and cyanotic CHD of each ACHD population.

**TRANSLATIONAL OUTLOOK:** The results of this study-level meta-analysis, based on a relatively large sample size, provide a foundation for future patient-level meta-analyses. Our findings also underscore the need for novel ASCVD-related research, focusing on ACHD-specific risk stratification and prevention strategies.

## Funding support and author disclosures

The authors have reported that they have no relationships relevant to the contents of this paper to disclose.
