# Supplementary material for: Atherosclerotic Risk Factor Prevalence in Adults With Congenital Heart Disease: A Meta-Analysis
Source: JACC Adv. 2024 Oct 19;3(11):101359. doi: 10.1016/j.jacadv.2024.101359 (PMC11533079; doi:10.1016/j.jacadv.2024.101359)

Supplemental Appendix:

Supplementary Tables:
Pages 3-5: Supplementary Table S1. PRISMA checklist as followed in the current systematic review and meta-analysis.
Pages 6-10: Supplementary Table S2. Definitions used for ACHD coding and for each ASCVD risk factor categorization in the included studies.
Pages 11-13: Supplementary Table S3. Quality assessment of the included studies using the questionnaire suggested by Munn et al.
Page 14: Supplementary Table S4. Demographic characteristics and ASCVD risk factor prevalence in ACHD populations of the studies included in this meta-analysis [data presented as mean (SD) or % (95% CIs)].
Pages 15-16: Supplementary Table S5. Meta-regression analyses on ASCVD risk factor prevalence.
Page 17: Supplementary Table S6. Risk ratios of ASCVD risk factors among ACHD and control populations.
Page 18: Supplementary Table S7. Comparison of extracted continuous data among ACHD and control populations [data presented as mean difference (95% CI)].
Page 19-20: Supplementary Table S8. Meta-regression analyses on the pooled risk ratios for ASCVD risk factor prevalence among ACHD and control populations.
Page 21: Supplementary Table S9. Assessment of the quality of evidence provided by the meta-analysis using the GRADE assessment tool.

Supplementary Figures:
Page 22: Supplementary Figure S1.
Full title. Scatter plot of the meta-regression analysis correlating hypertension (%) with age (years).
Short title. Meta-regression scatter plot of hypertension (%) with age (years).
Caption. Each point indicates the point estimate (%) of the respective included study.

Page 23: Supplementary Figure S2.
Full title. Scatter plot of the meta-regression analysis correlating dyslipidemia (%) with age (years).
Short title. Meta-regression scatter plot of dyslipidemia (%) with age (years).
Caption. Each point indicates the point estimate (%) of the respective included study.

Page 24: Supplementary Figure S3.
Full title. Scatter plot of the meta-regression analysis correlating dyslipidemia (%) with male gender (%).
Short title. Meta-regression scatter plot of dyslipidemia (%) with male gender (%).
Caption. Each point indicates the point estimate (%) of the respective included study.

Page 25: Supplementary Figure S4.
Full title. Scatter plot of the meta-regression analysis correlating hypertension (%) with cyanotic disease (%).
Short title. Meta-regression scatter plot of hypertension (%) with cyanotic disease (%).
Caption. Each point indicates the point estimate (%) of the respective included study.

Page 26: Supplementary Figure S5.

Full title. Scatter plot of the meta-regression analysis correlating smoking (%) with great congenital heart disease complexity (%).
Short title. Meta-regression scatter plot of smoking (%) with great congenital heart disease complexity (%).
Caption. Each point indicates the point estimate (%) of the respective included study.

Page 27: Supplementary Figure S6.
Full title. Funnel plots of the meta-analyses of proportions for the assessment of atherosclerotic risk factor prevalence in adults with congenital heart disease.
Short title. Funnel plots of the risk factor prevalence meta-analyses.
Caption. A. Hypertension, B. Diabetes mellitus, C. Dyslipidemia, D. Obesity, E. Smoking, F. Lack of regular exercise.

Page 28: Supplementary Figure S7.
Full title. Scatter plot of the meta-regression analysis correlating the risk ratio for hypertension with great congenital heart disease complexity (%).
Short title. Meta-regression scatter plot of hypertension risk with great congenital heart disease complexity (%).
Caption. Each point indicates the point estimate (log risk ratio) of the respective included study.

Page 29: Supplementary Figure S8.
Full title. Scatter plot of the meta-regression analysis correlating the risk ratio for diabetes mellitus with great congenital heart disease complexity (%).
Short title. Meta-regression scatter plot of diabetes mellitus risk with great congenital heart disease complexity (%).
Caption. Each point indicates the point estimate (log risk ratio) of the respective included study.

Page 30: Supplementary Figure S9.
Full title. Funnel plots of the meta-analyses of risk ratios for the comparison of atherosclerotic risk factor prevalence among adults with congenital heart disease and general population controls.
Short title. Funnel plots of the risk factor comparison meta-analyses.
Caption. A. Hypertension, B. Diabetes mellitus, C. Dyslipidemia, D. Obesity, E. Smoking, F. Lack of regular exercise.

Supplementary Tables:

Supplementary Table S1. PRISMA checklist as followed in the current systematic review and meta-analysis

**Title**	1	Identify the report as a systematic review.	*Page 1: "Title"*
**ABSTRACT**			
**Abstract**	2	See the PRISMA 2020 for Abstracts checklist.	*Page 2: "Abstract"*
**INTRODUCTION**			
**Rationale**	3	Describe the rationale for the review in the context of existing knowledge.	*Page 4: "Introduction."*
**Objectives**	4	Provide an explicit statement of the objective(s) or question(s) the review addresses.	*Page 4: "Hence, our systematic review ..risk factors."*
**METHODS**			
**Eligibility criteria**	5	Specify the inclusion and exclusion criteria for the review and how studies were grouped for the syntheses.	*Page 5: "Study selection – Eligibility criteria"*
**Information sources**	6	Specify all databases, registers, websites, organisations, reference lists and other sources searched or consulted to identify studies. Specify the date when each source was last searched or consulted.	*Page 5: "Search strategy and data sources"*
**Search strategy**	7	Present the full search strategies for all databases, registers and websites, including any filters and limits used.	*Page 5: "Search strategy and data sources"*
**Selection process**	8	Specify the methods used to decide whether a study met the inclusion criteria of the review, including how many reviewers screened each record and each report retrieved, whether they worked independently, and if applicable, details of automation tools used in the process.	*Page 5: "Study selection – Eligibility criteria"*
**Data collection process**	9	Specify the methods used to collect data from reports, including how many reviewers collected data from each report, whether they worked independently, any processes for obtaining or confirming data from study investigators, and if applicable, details of automation tools used in the process.	*Page 6: "Data extraction"*
**Data items**	10a	List and define all outcomes for which data were sought. Specify whether all results that were compatible with each outcome domain in each study were sought (e.g. for all measures, time points, analyses), and if not, the methods used to decide which results to collect.	*Pages 6-7: "Outcomes of interest" + "Data extraction"*
	10b	List and define all other variables for which data were sought (e.g. participant and intervention characteristics, funding sources). Describe any assumptions made about any missing or unclear information.	*Page 6: "Data extraction"*
**Study risk of bias assessment**	11	Specify the methods used to assess risk of bias in the included studies, including details of the tool(s) used, how many reviewers assessed each study and whether they worked independently, and if applicable, details of automation tools used in the process.	*Page 6: "Quality assessment"*
**Effect measures**	12	Specify for each outcome the effect measure(s) (e.g. risk ratio, mean difference) used in the synthesis or presentation of results.	*Pages 6-9: "Statistical analysis"*
**Synthesis methods**	13a	Describe the processes used to decide which studies were eligible for each synthesis (e.g. tabulating the study intervention characteristics and comparing against the planned groups for each synthesis (item #5)).	*Pages 6-9: "Statistical analysis"*

	13b	Describe any methods required to prepare the data for presentation or synthesis, such as handling of missing summary statistics, or data conversions.	*Pages 6-9: "Statistical analysis"*
	13c	Describe any methods used to tabulate or visually display results of individual studies and syntheses.	*Pages 6-9: "Statistical analysis"*
	13d	Describe any methods used to synthesize results and provide a rationale for the choice(s). If meta-analysis was performed, describe the model(s), method(s) to identify the presence and extent of statistical heterogeneity, and software package(s) used.	*Pages 6-9: "Statistical analysis"*
	13e	Describe any methods used to explore possible causes of heterogeneity among study results (e.g. subgroup analysis, meta-regression).	*Pages 6-9: "Statistical analysis"*
	13f	Describe any sensitivity analyses conducted to assess robustness of the synthesized results.	*Pages 6-9: "Statistical analysis"*
**Reporting bias assessment**	14	Describe any methods used to assess risk of bias due to missing results in a synthesis (arising from reporting biases).	*Page 7: "Statistical analysis": "Potential publication bias was evaluated through the Egger's test, the visual inspection of the relevant funnel plots and trim-and-fill analyses"*
**Certainty assessment**	15	Describe any methods used to assess certainty (or confidence) in the body of evidence for an outcome.	*Page 6: "Quality assessment"*
**RESULTS**			
**Study selection**	16a	Describe the results of the search and selection process, from the number of records identified in the search to the number of studies included in the review, ideally using a flow diagram.	*Page 9: "Search results and study selection" + Figure 1*
	16b	Cite studies that might appear to meet the inclusion criteria, but which were excluded, and explain why they were excluded.	*Figure 1+ Tables 1,2*
**Study characteristics**	17	Cite each included study and present its characteristics.	*Tables 1 and 2*
**Risk of bias in studies**	18	Present assessments of risk of bias for each included study.	*Supplementary Table S3 +Page 9: "Quality assessment and risk of bias"*
**Results of individual studies**	19	For all outcomes, present, for each study: (a) summary statistics for each group (where appropriate) and (b) an effect estimate and its precision (e.g. confidence/credible interval), ideally using structured tables or plots.	*Pages 9-13*
**Results of syntheses**	20a	For each synthesis, briefly summarise the characteristics and risk of bias among contributing studies.	*Pages 9-13*
	20b	Present results of all statistical syntheses conducted. If meta-analysis was done, present for each the summary estimate and its precision (e.g. confidence/credible interval) and measures of statistical heterogeneity. If comparing groups, describe the direction of the effect.	*Pages 9-13+ Suppl Tables*
	20c	Present results of all investigations of possible causes of heterogeneity among study results.	*Pages 9-13+ Suppl Tables*
	20d	Present results of all sensitivity analyses conducted to assess the robustness of the synthesized results.	*Pages 9-13+ Suppl Tables*
**Reporting biases**	21	Present assessments of risk of bias due to missing results	*Pages 9-13+ Suppl Tables*

		(arising from reporting biases) for each synthesis assessed.	
**Certainty of evidence**	22	Present assessments of certainty (or confidence) in the body of evidence for each outcome assessed.	*Page 13: "GRADE appraisal of the quality of evidence" + Suppl Table S9*
**DISCUSSION**			
**Discussion**	23a	Provide a general interpretation of the results in the context of other evidence.	*Pages 13-19: "Discussion"*
	23b	Discuss any limitations of the evidence included in the review.	*Pages 18-19: "Strengths and Limitations*
	23c	Discuss any limitations of the review processes used.	*Pages 18-19: "Strengths and Limitations*
	23d	Discuss implications of the results for practice, policy, and future research.	*Pages 18-19: "Strengths and Limitations + Page 19: "Conclusions"*
**OTHER INFORMATION**			
**Registration and protocol**	24a	Provide registration information for the review, including register name and registration number, or state that the review was not registered.	*Page 5: "Our systematic review and meta-analysis was registered on the PROSPERO registry (CRD42023490437)."*
	24b	Indicate where the review protocol can be accessed, or state that a protocol was not prepared.	*Page 5: "Our systematic review and meta-analysis was registered on the PROSPERO registry (CRD42023490437)."*
	24c	Describe and explain any amendments to information provided at registration or in the protocol.	*Not applicable.*
**Support**	25	Describe sources of financial or non-financial support for the review, and the role of the funders or sponsors in the review.	*Page 1: "Funding support and author disclosures:"*
**Competing interests**	26	Declare any competing interests of review authors.	*Page 1: "Funding support and author disclosures:"*
**Availability of data, code and other materials**	27	Report which of the following are publicly available and where they can be found: template data collection forms; data extracted from included studies; data used for all analyses; analytic code; any other materials used in the review.	*Page 18: "Data availability"*

Supplementary Table S2. Definitions used for ACHD coding and for each ASCVD risk factor categorization in the included studies.

Study	ACHD	Risk factors					
		HTN	DM	DLD	Ob	Smok	Lack of Exer
Moons 2006	History of ACHD	Anti-HTN medication	Anti-DM medication	n.a.	BMI > 30 kg/m^2	By self-report	By self-report (lack of exercise< 3 times per week)
Vriend 2006	ICD-10	SBP>150	n.a.	n.a.	n.a.	By self-report	n.a.
Martínez-Quintana 2010	Post-surgery pts	n.a.	HbA1c > 6.5	n.a.	n.a.	n.a.	n.a.
Duffels 2010	Cyanotic (transcutaneous oxygen saturation <90% at rest)	DBP >90 mmHg, SBP >140 mmHg, or use of anti-HTN medication	n.a.	n.a.	n.a.	By self-report	n.a.
Zomer 2012	History of CHD	n.a.	By self-report	n.a.	BMI > 30 kg/m^2	By self-report	By self-report: Sports participation: ≥ 1 h/week participation in sports (walking/ bicycling not included)
Ohuchi 2014	History of unrepaired CHD after biventricular repair and Fontan	n.a.	n.a.	n.a.	n.a.	n.a.	n.a.
Dellborg 2015	ICD 9-10	Anti-HTN medication	n.a.	Lipid-lowering medication	n.a.	By self-report	By self-report: Never or <1 times/week, Regular 1 – 2 times/week, High-Regular 3 – 5 times/week
Moon 2015	History of Cyanotic CHD (peripheral O2 <93% in room air, unrepaired intra- or extracardiac shunt, and severe PH diagnosed by echocardiography or cardiac catheterization)	Anti-HTN medication, clinically diagnosed with HT, or had either an SBP ≥140 mmHg or a DBP ≥90 mmHg.	Anti-DM medication, clinical diagnosis of DM, or an FBS level>126 mg/dL	Medication for hypercholesterolemia, TC>200 mg/dL, or LDL-C > 130 mg/dL	BMI ≥30.0 kg/m^2	By self-report	By self-report: "Exercise" meant moderate activity for >30 min five days per week, intense activity for >20 min three days per week) or walking for >30

	and surgically corrected ACHD without cyanosis after total correction via cardiac surgery.						min a day for more than five days per week
Sandberg 2015	History of CHD	n.a.	n.a.	n.a.	BMI ≥ 30 kg/m^2	n.a.	Self-reported level of physical exercise (no exercise, <3 h/week, >3 h/week)
Madsen 2016	CHD based on review of inpatient and outpatient medical records by an experienced physician-later translated to the ICD-10	n.a.	ICD codes for DM or Anti-DM medication	n.a.	n.a.	n.a.	n.a.
Caruana 2016	History of ACHD	n.a.	n.a.	n.a.	n.a.	By self-report (Smoking tobacco daily/ occasionally at the time of the study)	By self-report: Low versus moderate&high intensity physical activity levels, following the instruments used during EHIS 2008
Deen 2016	History of ACHD	SBP ≥130 mmHg or DBP ≥85 mmHg or Anti-HTN medication	Fasting hyperglycemia ≥100 mg/dL or previously diagnosed DM	Hypertriglyceridemia: ≥150 mg/dL or treatment for this lipid abnormality, Reduced HDL: <40 mg/dL in males <50 mg/dL in females or treatment for this lipid abnormality	BMI ≥30 kg/m^2	n.a.	n.a.
Fedchenko 2019	Diagnosis of CHD as a principal or contributory	Codes 401–405 (ICD-8 and ICD-9) or I10–I15 (ICD-10)	Code 250 (ICD-8 and ICD-9) or codes	n.a.	n.a.	n.a.	By self-report: ESC recommendations

	diagnosis (ICD 8-10)		E10–E14 (ICD-10)				on physical exercise (150 minute/week of moderate physical activity or 75 minute/week of intense physical activity, or a combination of these)
Trojnarska 2017	Cyanotic CHD with arterial blood oxygen saturation <92%	n.a.	n.a.	n.a.	n.a.	n.a.	n.a.
Lerman 2017	ICD-9	n.a.	n.a.	n.a.	BMI> 30 kg/m^2	n.a.	n.a.
Flannery 2018	History of CHD	Diagnosis of HTN	History of DM	Statin treatment	BMI> 30 kg/m^2	By self-report	n.a.
Pickard 2018	Repaired CoA (ICD-9)	History of HTN	History of DM	History of dyslipidemia	n.a.	By self-report	n.a.
Tarp 2018	Cyanotic CHD defined as the presence of a congenital heart defect with a right-to-left or bidirectional shunt, with resting systemic O2 <92% and/or <87% during exercise	History of HTN	History of DM	History of dyslipidemia	BMI ≥30 kg/m^2	n.a.	By self-report (regular weekly exercise)
Saha 2019	CHD defined as any structural cardiac abnormality, inclusive of BAV, present in the heart or adjoining great vessels from birth in the absence of syndromic illness (i.e., syndromes characterized by extra-cardiac or neurocognitive	Hospital diagnoses occurring anytime up to time of enrollment, self-reported diagnoses, self-reported use of anti-HTN medication, and/or BP >140/90	Type 1 and 2 DM occurring anytime up to enrollment, self-reported history of DM, and/or usage of insulin	Primary and secondary hospital diagnoses occurring anytime up to time of enrollment, self-reported diagnoses, and/or usage of statin therapy	BMI >30 kg/m^2	By self-report	Self-report of number of days of moderate or vigorous intensity exercise per week, and dichotomized as low or high based on satisfaction of WHO recommendations for >=5 days/week of moderate intensity exercise or >=3 days/week of

	manifestations in addition to cardiac malformations) ICD 9-10						vigorous intensity exercise
Martínez-Quintana 2019	CHD verified by echocardiography, CMR and/or cardiac catheterization	Office SBP ≥140 mm Hg and/or DBP ≥90 mm Hg or anti-HTN medication	FBG >126 mg/dL or anti-DM medication	LDL>130 mg/dL or under statin treatment	n.a.	By self-report	n.a.
Krishnamurthy 2019	Patients with CoA and a coronary CT angiography or chest CT with interpretable coronary information	Diagnosis of HTN	Diagnosis of DM	n.a.	BMI ≥30 kg/m^2	By self-report	n.a.
Tarp 2019	Cyanotic CHD defined as a congenital heart defect resulting in oxygen saturation at rest of <92%	History of HTN	n.a.	n.a.	n.a.	n.a.	n.a.
Zaqout 2019	History of CHD	Diagnosis of HTN according to ESC	Diagnosis of DM according to ESC	Diagnosis of dyslipidemia according to ESC	BMI ≥30 kg/m^2	By self-report	n.a.
Gales 2020	ICD-9	History of HTN	History of DM	History of dyslipidemia	n.a.	By self-report	n.a.
Sandberg 2013	Congenital AS or AR diagnosis	n.a.	n.a.	n.a.	n.a.	By self-report	EQ-5D self-report questionnaire, self-reported exercise time
Buys 2013	History of CoA	n.a.	n.a.	n.a.	BMI > 30 kg/m^2	n.a.	By self-report (sedentary, active or moderately active and a vigorously active lifestyle)
Lanz 2015	ICD-9/10	ICD-9/10	ICD-9/10	ICD-9/10	ICD-9/10	ICD-9/10	n.a.
Karsenty 2015	CHD diagnosis, Bethesda criteria	n.a.	n.a.	n.a.	n.a.	By self-report	Self-reported (WHO-MONICA Optional Study of Physical Activity Questionnaire)

Muller 2017	CHD diagnosis, ACC criteria	n.a.	n.a.	n.a.	n.a.	n.a.	IPAQ
Larsson 2019	ICD-9/10	n.a.	n.a.	n.a.	n.a.	By self-report	Actiheart accelerometer and IPAQ
Malavazos 2019	CHD diagnosis, Bethesda classification	n.a.	n.a.	n.a.	BMI ≥ 30 kg/m^2	n.a.	n.a.
Holbein 2020	CHD diagnosis	n.a.	n.a.	n.a.	n.a.	By self-report	Self-reported (Health-Behavior Scale-Congenital Heart Disease)
Lubert 2021	Fontan circulation	n.a.	n.a.	n.a.	n.a.	n.a.	n.a.
Fox 2021	CHD diagnosis, Classification by Warnes et al., 2008	n.a.	n.a.	n.a.	n.a.	By self-report	n.a.
Umapathi 2022	SNOMED-CT diagnostic codes and 2018 AHA guidelines	International Diabetes Federation's criteria of MetS	International Diabetes Federation's criteria of MetS	International Diabetes Federation's criteria of MetS	BMI ≥ 30 kg/m^2	By self-report	n.a.
Garcia Cruz 2023	2020 European Society of Cardiology Guidelines for the management of adult CHD	SBP≥ 130 or DBP ≥ 80 mmHg	Fasting plasmatic glucose >126 mg/dL or a HBA_1c ≥ 6.5%	ICD-10	BMI > 30 kg/m^2	By self-report: >100 cigarettes in lifetime and current smoker	By self-report: 30 minutes daily for at least 5 days a week for moderate-intensity aerobic physical activity
Bjork 2024	CHD diagnosis, hierarchic classification by Botto (modified by Liu)	n.a.	ICD-8/9/10	n.a.	n.a.	n.a.	n.a.
Kowalik 2024	CHD diagnosis, ESC guidelines	Anti-HTN treatment	Hypoglycemic medication or American Diabetes Association criteria	Lipid-lowering therapy or European Society of Cardiology guidelines	BMI ≥ 30 kg/m^2	n.a.	n.a.

Supplementary Table S3. Quality assessment of the included studies using the questionnaire suggested by Munn et al

Author	Year	1. Sample frame appropriateness	2. Appropriateness of study participants' sampling	3. Sample size adequacy	4. Detail of study setting's description	5. Sufficient sample coverage in data analysis	6. Validity of the identifying methods	7. Reliability of the condition's measurement	8. Appropriateness of the statistical analysis	9. Response rate adequacy
Moons et al	2005	Y	Y	Y	Y	Y	Y	U	Y	N
Vriend et al	2006	Y	Y	U	Y	Y	Y	Y	Y	U
Martinez-Quintana et al	2010	Y	Y	U	Y	Y	Y	Y	Y	N
Duffels et al	2010	Y	Y	N	Y	Y	Y	Y	Y	N
Luijendijk et al	2014	Y	Y	U	Y	Y	Y	Y	Y	N
Ohuchi et al	2014	Y	U	U	Y	Y	Y	Y	Y	N
Dellborg et al	2015	Y	Y	U	N	U	Y	Y	Y	N
Moon et al	2015	Y	Y	U	Y	Y	Y	Y	Y	N
Sandberg et al	2015	Y	Y	Y	Y	Y	Y	Y	Y	N
Madsen et al	2016	Y	Y	Y	Y	U	U	U	Y	N
Caruana et al	2016	Y	Y	U	Y	U	N	Y	Y	Y
Deen et al	2016	Y	Y	U	Y	U	Y	Y	Y	N
Trojnarska et al	2017	Y	Y	U	Y	Y	Y	Y	Y	Y
Fedchenko et al	2017	Y	Y	Y	Y	Y	Y	Y	Y	N
Flannery et al	2018	Y	Y	U	Y	Y	Y	Y	Y	N
Pickard et al	2018	Y	Y	U	U	Y	Y	U	Y	U
Saha et al	2019	Y	Y	Y	N	Y	N	Y	Y	N
Martínez-Quintana et al	2018	Y	Y	U	Y	Y	Y	Y	Y	N
Gales et al	2020	Y	Y	U	N	Y	Y	Y	Y	N
Krishnamurthy et al	2019	Y	Y	U	U	Y	Y	Y	Y	Y
Tarp et al	2018	Y	Y	N	Y	Y	Y	Y	Y	N
Lerman et al	2017	Y	Y	Y	Y	Y	Y	Y	Y	N
Zaqout et al	2019	Y	Y	U	Y	Y	N	Y	Y	Y
Zomer et al	2012	Y	Y	Y	Y	Y	N	Y	Y	N
Bjork et al	2024	Y	Y	Y	Y	Y	Y	Y	Y	N
Larsson et al	2019	Y	Y	N	Y	Y	N	Y	Y	N
Lubert et al	2021	Y	Y	U	Y	Y	Y	Y	Y	N
Malavazos et al	2019	Y	Y	Y	Y	Y	Y	Y	Y	N
Umapathi et al	2022	Y	Y	Y	Y	Y	Y	Y	Y	N
Engelfriet et al	2007	Y	Y	Y	Y	U	N	Y	Y	U
Giannakoulas et al	2009	Y	Y	U	Y	Y	Y	Y	Y	N
Roifman et al	2012	Y	Y	U	N	U	Y	Y	Y	N
Lui et al	2018	Y	Y	U	N	U	Y	N	Y	N
Bokma et al	2018	Y	Y	N	Y	Y	Y	Y	Y	Y
Bauer et al	2018	Y	Y	U	Y	Y	Y	Y	Y	N

**Egbe et al**	2019	Y	Y	U	N	Y	Y	Y	Y	N
**Johnson et al**	2019	Y	Y	N	Y	Y	Y	N	Y	N
**Fedchenko et al**	2019	Y	Y	N	Y	Y	Y	Y	Y	Y
**Egbe et al**	2019	Y	Y	U	Y	Y	Y	Y	Y	Y
**Murakami et al**	2021	Y	Y	N	Y	Y	Y	Y	Y	N
**Meijs et al**	2021	Y	Y	U	Y	Y	Y	Y	Y	N
**Misra et al**	2022	Y	Y	U	Y	U	Y	Y	Y	N
**Jepson et al**	2022	Y	Y	Y	N	Y	Y	Y	Y	N
**Egbe et al**	2022	Y	N	Y	N	Y	Y	Y	Y	N
**Fyfe et al**	2005	Y	U	U	Y	Y	Y	Y	Y	N
**Kuijpers et al**	2020	Y	Y	Y	N	U	Y	Y	Y	N
**Zaidi et al**	2011	Y	Y	U	N	Y	Y	Y	Y	N
**Freud et al**	2015	Y	Y	U	Y	Y	Y	Y	Y	N
**Chung et al**	2016	Y	Y	U	N	Y	Y	Y	Y	N
**Brida et al**	2017	Y	Y	Y	Y	Y	Y	Y	Y	Y
**Jackson et al**	2020	Y	Y	Y	N	Y	Y	Y	Y	N
**Tutarel et al**	2014	Y	Y	U	Y	Y	Y	Y	Y	N
**Buys et al**	2013	Y	Y	U	Y	Y	U	Y	Y	N
**Fox et al**	2021	Y	Y	Y	N	Y	N	Y	Y	N
**Garcia Cruz et al**	2023	Y	Y	Y	Y	Y	Y	Y	Y	N
**Holbein et al**	2020	Y	Y	Y	U	Y	N	Y	Y	N
**Karsenty et al**	2015	Y	Y	U	Y	Y	N	Y	Y	N
**Kowalik et al**	2024	Y	Y	U	Y	Y	Y	Y	Y	N
**Lanz et al**	2015	Y	Y	U	N	Y	Y	Y	Y	N
**Muller et al**	2017	Y	Y	U	N	Y	N	Y	Y	N
**Sandberg et al**	2013	Y	Y	U	N	Y	N	Y	Y	N

Abbreviations: Y, yes; N, no; U, unclear

Questions answered for the completion of the questionnaire:

1. Was the sample frame appropriate to address the target population?

Y: ACHD patients (diagnosis settled by expert physicians)
N: Non-ACHD patients
U: Unclear if ACHD patients (ICD-10 based or other)

2. Were study participants sampled in an appropriate way?

Y: ACHD patients consecutively or randomly recruited
N: ACHD patients non-consecutively or non-randomly recruited
U: ACHD patients unclear if consecutively or randomly recruited

3. Was the sample size adequate?

Y: Evidence that the authors conducted a sample size calculation to determine an adequate sample size OR sample size ≥1000
N: No-evidence that the authors conducted a sample size calculation to determine an adequate sample size OR sample size <100
U: No-evidence that the authors conducted a sample size calculation to determine an adequate sample size OR sample size 100-999

4. Were the study subjects and the setting described in detail?

Y: Baseline characteristics and ACHD subtypes/severity clearly described
N: Baseline characteristics and ACHD subtypes/severity non-clearly described
U: Baseline characteristics or ACHD subtypes/severity non-clearly described

5. Was the data analysis conducted with sufficient coverage of the identified sample?
Y: Response rate ≥90%
N: Response rate <75%
U: Response rate not reported

6. Were valid methods used for the identification of the condition?
Y: Most of the risk factors are properly measured/defined according to established guidelines (e.g., not self-reported)
N: Most of the risk factors are not properly measured/defined according to established guidelines (e.g., self-reported)
U: Equal number of risk factors are properly measured/defined according to established guidelines (e.g., not self-reported)

7. Was the condition measured in a standard, reliable way for all participants?
Y: Risk factors were measured in the same way (observers, methodology of measurement) for all participants
N: Risk factors were not measured in the same way (observers, methodology of measurement) for all participants
U: Unclear if the risk factors were measured in the same way (observers, methodology of measurement) for all participants

8. Was there appropriate statistical analysis?
Y: Numerators, denominators or percentages with confidence intervals reported
N: Numerators, denominators or percentages with confidence intervals not reported
U: Numerators, denominators or percentages with confidence intervals not clearly reported

9. Was the response rate adequate, and if not, was the low response rate managed appropriately?
Y: Low response rate is adequately discussed and addressed (including comparison of responders vs non-responders) and reasons for non-response appear to be unrelated to the outcome measured
N: Low response rate is not adequately discussed and addressed (including comparison of responders vs non-responders) and reasons for non-response appear to be related to the outcome measured
U: Low response rate is unclearly discussed and addressed (including comparison of responders vs non-responders) and it is unclear whether reasons for non-response are related to the outcome measured

Supplementary Table S4. Demographic characteristics and ASCVD risk factor prevalence in ACHD populations of the studies included in this meta-analysis [data presented as mean (SD) or % (95% CIs)]

Variable	Main analysis (62 studies in total)	Subgroup of 100% cyanotic populations (5 studies in total)	Subgroup of 100% non-cyanotic populations (14 studies in total)	Subgroup of 100% CoA populations (8 studies in total)
**Male gender, %**	52 (50-54)<br>*55 studies*	52 (47-57)<br>*5 studies*	55 (50-59)<br>*14 studies*	58 (55-61)<br>*8 studies*
**Mean age, years**	38.5 (18.6)<br>*54 studies*	35.5 (16.9)<br>*5 studies*	42.6 (17.1)<br>*14 studies*	36.8 (16)<br>*8 studies*
**Mean BMI, kg/m²**	24.5 (4.9)<br>*32 studies*	22.8 (6.2)<br>*4 studies*	24.8 (5.4)<br>*10 studies*	24.6 (5.4)<br>*6 studies*
**Cyanotic status, %**	8 (8-9)<br>*32 studies*	100	0	0
**Great CHD complexity, %**	11 (4-29)<br>*52 studies*	100	NA	0
**Hypertension, %**	33 (26-40)<br>*34 studies*	5 (2-10)<br>*3 studies*	47 (37-58)<br>*11 studies*	57 (49-64)<br>*7 studies*
**Diabetes mellitus, %**	7 (5-9)<br>*33 studies*	NA	7 (4-11)<br>*11 studies*	5 (3-8)<br>*6 studies*
**Dyslipidemia, %**	17 (11-25)<br>*27 studies*	NA	19 (10-33)<br>*10 studies*	14 (7-25)<br>*5 studies*
**Obesity, %**	18 (14-22)<br>*26 studies*	NA	18 (11-29)<br>*5 studies*	NA
**Current smoking, %**	12 (9-14)<br>*40 studies*	5 (2-10)<br>*3 studies*	11 (7-17)<br>*12 studies*	14 (8-24)<br>*6 studies*
**Lack of regular exercise, %**	54 (45-63)<br>*18 studies*	NA	58 (48-68)<br>6 studies	NA

ACHD, adults with congenital heart disease; ASCVD, atherosclerotic cardiovascular disease; BMI, body mass index; CHD, congenital heart disease; CI, confidence intervals; NA, not applicable; SD, standard deviation.
Means and standard deviations (SDs) represent pooled estimates from continuous variables (i.e., age and BMI) across all studies. Proportions (in %) are accompanied by 95% confidence intervals (CIs), derived from meta-proportion analyses conducted using the 'metafor' and 'meta' R packages. These meta-proportion analyses pooled categorical variables (e.g., male gender, cyanotic status, CHD complexity, hypertension, diabetes mellitus, dyslipidemia, obesity, smoking, and lack of regular exercise) based on data from the included studies.

Supplementary Table S5. Meta-regression analyses on ASCVD risk factor prevalence

Covariate	Hypertension	Diabetes mellitus	Dyslipidemia	Obesity	Smoking	Lack of regular Exercise
**Publication year**	R^2=0.00% β=0.0071 CIs adj= (-0.0088, 0.0230) se=0.0065	R^2=10.13% β=0.0068 CIs adj= (-0.0025, 0.0156) se=0.0038	R^2=16.34% β=0.0197 CIs adj= (-0.0016, 0.0413) se=0.0087	R^2=0.00% β=0.0013 CIs adj= (-0.0114, 0.0140) se=0.0052	R^2=0.00% β=-0.0015 CIs adj= (-0.0084, 0.0054) se=0.0028	R^2=0.00% β=-0.0077 CIs adj= (-0.0358, 0.0204) se=0.0115
**Sample size**	R^2=0.00% β<-0.0001 CIs adj= (-0.0003, 0.0001) se<0.0001	R^2=0.00% β<0.0001 CIs adj= (-0.0002, 0.0002) se<0.0001	R^2=0.00% β<0.0001 CIs adj= (-0.0002, 0.0002) se<0.0001	R^2=0.00% β<0.0001 CIs adj= (-0.0002, 0.0002) se<0.0001	R^2=0.00% β<-0.0001 CIs adj= (-0.0002, 0.0002) se<0.0001	R^2=0.00% β<0.0001 CIs adj= (-0.0002, 0.0002) se<0.0001
**Age**	**R^2=19.77% β=0.0075 CIs adj= (0.0004, 0.0146) se=0.0029**	R^2=0% β=0.0011 CIs adj= (-0.0026, 0.0048) se=0.0015	**R^2=44.30% β=0.0107 CIs adj= (0.0041, 0.0173) se=0.0027**	R^2=5.57% β=0.0039 CIs adj= (-0.0025, 0.0098) se=0.0026	R^2=0.00% β=0.0018 CIs adj= (-0.0021, 0.0057) se=0.0016	R^2=0.00% β=-0.0022 CIs adj= (-0.0142, 0.0098) se=0.0049
**Male%**	R^2=0.00% β=-0.0001 CIs adj= (-0.0101, 0.0099) se=0.0041	R^2=5.89% β=-0.0044 CIs adj= (-0.0103, 0.0015) se=0.0024	**R^2=19.59% β=-0.0100 CIs adj= (-0.0198, -0.0002) se=0.0040**	R^2=0.00% β=-0.0014 CIs adj= (-0.0102, 0.0074) se=0.0036	R^2=0.00% β=0.0017 CIs adj= (-0.0054, 0.0088) se=0.0029	R^2=0.00% β=0.0010 CIs adj= (-0.0117, 0.0137) se=0.0052
**Cyanotic%**	**R^2=22.40% β=-0.0044 CIs adj= (-0.0088, -0.00001) se=0.0018**	R^2= 0.00% β<0.0001 CIs adj= (-0.0017, 0.0017) se=0.0007	R^2=0.00% β=-0.0011 CIs adj= (-0.0050, 0.0028) se=0.0016	R^2=0.00% β=-0.0013 CIs adj= (-0.0052, 0.0026) se=0.0016	R^2=0.00% β=-0.0005 CIs adj= (-0.0027, 0.0017) se=0.0009	NA (9 studies)

**Great CHD complexity%**	R^2=10.99% β=-0.0030 CIs adj= (-0.0067, 0.0007) se=0.0015	R^2=11.58% β=0.0009 CIs adj= (-0.0008, 0.0026) se=0.0007	R^2=0.00% β=-0.0012 CIs adj= (-0.0044, 0.0020) se=0.0013	R^2=0.00% β=0.0004 CIs adj= (-0.0023, 0.0031) se=0.0011	**R^2=25.67% β=-0.0016 CIs adj= (-0.0033, -0.0001) se=0.0007**	R^2=5.73% β=-0.0022 CIs adj= (-0.0066, 0.0022) se=0.0018

R^2: amount of heterogeneity accounted for; β=coefficient;
ASCVD, atherosclerotic cardiovascular disease; CI adj, Confidence intervals after adjustment; NA, not applicable; se, standard error
Cells in bold represent the statistically significant outcomes of these meta-regression analyses.

Supplementary Table S6. Risk ratios of ASCVD risk factors among ACHD and control populations

Analysis	Hypertension	Diabetes mellitus	Dyslipidemia	Obesity	Smoking	Lack of regular exercise
**Main analysis**	RR=1.02 (0.92 - 1.12), I^2=94% 16 studies	**RR=1.30 (1.09 - 1.55) I^2=92% 17 studies**	RR=0.99 (0.76 - 1.27), I^2=98% 11 studies	RR=1.01 (0.92 - 1.12), I^2=85% 12 studies	**RR=0.67 (0.57 - 0.80), I^2=89% 18 studies**	RR=1.16 (0.98 -1.37), I^2=97% 8 studies
**Only age-, sex-matched studies**	RR=1.03 (0.86 - 1.24), I^2=93% 10 studies	**RR=1.32 (1.00 - 1.74), I^2=88% 10 studies**	RR=0.96 (0.69 - 1.33), I^2=91% 6 studies	RR=0.98 (0.80 - 1.20), I^2=86% 5 studies	**RR=0.62 (0.47 - 0.80), I^2=79% 11 studies**	RR=1.26 (0.90 - 1.76), I^2=98% 5 studies
**Without studies having an inclusion criterion of comorbid CVD**	RR=1.06 (0.94 - 1.21), I^2=94% 12 studies	**RR=1.49 (1.24 - 1.78), I^2=86% 14 studies**	RR=1.01 (0.70 - 1.45), I^2=99% 8 studies	RR=1.01 (0.88 - 1.16), I^2=87% 10 studies	**RR=0.62 (0.51 - 0.76), I^2=90% 15 studies**	RR=1.19 (0.97 - 1.45), I^2=97% 7 studies
**100% cyanotic populations**	**RR=0.18 (0.06 - 0.53), I^2=19% 3 studies**	NA	NA	NA	RR=0.42 (0.11 - 1.67), I^2=67% 3 studies	NA
**100% non-cyanotic populations**	**RR=1.35 (1.04 - 1.74), I^2=92% 5 studies**	RR=1.29 (0.93 - 1.78), I^2=81% 4 studies	RR=1.42 (0.84 - 2.40), I^2=98% 3 studies	RR=1.04 (0.71 - 1.52), I^2=92% 3 studies	RR=0.96 (0.75 - 1.23), I^2=59% 5 studies	NA
**Without very large studies (sample size >10,000)**	RR=0.95 (0.80 - 1.13), I^2=92% 13 studies	RR=1.00 (0.61 – 1.63), I^2=89% 12 studies	RR=0.83 (0.60 - 1.14), I^2=93% 8 studies	RR=0.99 (0.85 – 1.14), I^2=82% 9 studies	**RR=0.60 (0.50 - 0.71), I^2=74% 14 studies**	RR=1.19 (0.96 – 1.48), I^2=97% 7 studies

ACHD, adults with congenital heart disease; ASCVD, atherosclerotic cardiovascular disease; CHD, congenital heart disease; NA, not applicable. Cells in bold represent the statistically significant outcomes of these meta-analyses ($p<0.05$).

Supplementary Table S7. Comparison of extracted continuous data among ACHD and control populations [data presented as mean difference (95% CI)]

Variable	Main analysis	Only age-, sex-matched studies	Without studies having an inclusion criterion of comorbid CVD	100% cyanotic populations	100% non-cyanotic patients
**Age (years)**	-0.82 (-1.75, 0.11), I^2=93% 24 studies	0.19 (-0.20, 0.57), I^2=0% 13 studies	-0.43 (-1.45, 0.62), I^2=92% 21 studies	-0.82 (-4.15, 2.50), I^2=45% 4 studies	-1.40 (-4.01, 1.25), I^2=85% 7 studies
**Body mass index (kg/m^2)**	**-0.76 (-1.07, -0.44), I^2=76% 17 studies**	**-0.81 (-1.25, -0.32), I^2=65% 10 studies**	**-0.83 (-1.24, -0.47), I^2=80% 15 studies**	**-0.97 (-1.86, -0.09), I^2=0% 3 studies**	-0.85 (-2.39, 0.63), I^2=86% 4 studies
**Fasting blood glucose (mg/dl)**	0.43 (-2.57, 3.44), I^2=85% 7 studies	-0.38 (-5.14, 4.39), I^2=80% 4 studies	NA	2.40 (-3.30, 8.10), I^2=78% 3 studies	NA
**Systolic Blood Pressure/24h (mmHg)**	0.38 (-3.03, 3.79), I^2=95% 9 studies	0.24 (-6.19, 6.66), I^2=97% 6 studies	0.49 (-4.40, 5.38), I^2=96% 8 studies	**-8.44 (-15.71, -1.16), I^2=86% 4 studies**	6.43 (-2.58, 15.43), I^2=98% 4 studies
**Diastolic Blood Pressure/24h (mmHg)**	-0.80 (-3.71, 2.11), I^2=94% 7 studies	-1.82 (-6.46, 2.82), I^2=93% 4 studies	NA	-4.22 (-8.82, 0.38), I^2=86% 4 studies	**2.73 (0.49, 4.98), I^2=73% 3 studies**
**Glycated hemoglobin (HbA1c, %)**	0.13 (-0.90, 1.15), I^2=98% 3 studies	NA	NA	NA	NA
**Serum triglycerides (mg/dl)**	2.80 (-4.83, 10.42), I^2=64% 8 studies	3.50 (-11.65, 19.85), I^2=84% 5 studies	NA	10.86 (-7.61, 29.33), I^2=68% 3 studies	NA
**Total cholesterol (mg/dl)**	**-19.22 (-25.70, -12.75), I^2=85% 11 studies**	**-17.38 (-23.45, -10.67), I^2=42% 7 studies**	NA	**-17.61 (-26.40, -8.82), I^2=38% 4 studies**	-12.63 (-30.45, 9.23), I^2=85% 4 studies
**Low-density lipoprotein (mg/dl)**	**-9.62 (-15.16, -4.08), I^2=82% 10 studies**	**-11.79 (-15.93, -7.27), I^2=44% 6 studies**	NA	-5.08 (-10.87, 0.71), I^2=0% 4 studies	NA
**High-density lipoprotein (mg/dl)**	**-8.73 (-12.30, -5.15), I^2=85% 11 studies**	**-8.67 (-11.55, -3.56), I^2=88% 7 studies**	NA	**-9.35 (-15.51, -3.19), I^2=86% 4 studies**	-1.99 (-8.82, 4.70), I^2=80% 4 studies
**Carotid intima media thickness (mm)**	0.06 (-0.01, 0.12), I^2=94% 5 studies	0.05 (-0.03, 0.12), I^2=95% 4 studies	NA	0.01 (-0.04, 0.07), I^2=80% 3 studies	NA

ACHD, adults with congenital heart disease; CVD, cardiovascular disease
Cells in bold represent the statistically significant outcomes of these meta-analyses ($p<0.05$).

Supplementary Table S8. Meta-regression analyses on the pooled risk ratios for ASCVD risk factor prevalence among ACHD and control populations.

Outcome assessed	Meta-regression for publication year	Meta-regression for sample size	Meta-regression for mean age of ACHD	Meta-regression for % of males	Meta-regression for % of cyanotic CHD	**Meta-regression % of gre CHD complexi**
**Hypertension**	p=0.63 R^2=0.00% β=0.0123 CIs adj= (-0.0496, 0.0742) se=0.0253	p=0.71 R^2=0.00% β<0.0001 CIs adj= (-0.0002, 0.0002) se<0.0001	p=0.48 R^2=0.00% β=0.0057 CIs adj= (-0.0200, 0.0314) se=0.0105	p=0.36 R^2=0.00% β=0.0126 CIs adj= (-0.0236, 0.0488) se=0.0148	NA	**p=0.02 R^2=14.48 β=-0.01 CIs adj (-0.0183 -0.0018 se=0.00**
**Diabetes mellitus**	p=0.74 R^2=0.00% β=-0.0143 CIs adj= (-0.1109, 0.0823) se=0.0437	p=0.45 R^2=0.00% β<0.0001 CIs adj= (-0.0002, 0.0002) se<0.0001	p=0.99 R^2=0.00% β<-0.0001 CIs adj= (-0.0002, 0.0002) se<0.0001	p=0.95 R^2=0.00% β=-0.0015 CIs adj= (-0.0167, 0.0140) se=0.0039	NA	**p<0.01 R^2=44.8 β=0.016 CIs adj (0.0057, 0.0 se=0.00**
**Dyslipidemia**	p=0.69 R^2=0.00% β=-0.0350 CIs adj= (-0.2531, 0.1831) se=0.0890	p=0.60 R^2=0.00% β<0.0001 CIs adj= (-0.0002, 0.0002) se<0.0001	p=0.41 R^2=0.00% β=0.0128 CIs adj= (-0.0207, 0.0464) se=0.0137	p=0.73 R^2=0.00% β=0.0072 CIs adj= (-0.0369, 0.0513) se=0.0180	NA	NA
**Obesity**	p=0.29 R^2=0.00% β=-0.0157 CIs adj= (-0.0519, 0.0205) se=0.0148	p=0.57 R^2=0.00% β<0.0001 CIs adj= (-0.0002, 0.0002) se<0.0001	p=0.44 R^2=0.00% β=0.0128 CIs adj= (-0.0256, 0.0512) se=0.0157	p=0.45 R^2=0.00% β=0.0079 CIs adj= (-0.0178, 0.0336) se=0.0105	NA	NA

**Smoking**	p=0.69<br>R^2=0.00%<br>β=-0.0124<br>CIs adj=<br>(-0.0877,<br>0.0630)<br>se=0.0308	p=0.13<br>R^2=12.68%<br>β<0.0001<br>CIs adj=<br>(-0.0002, 0.0002)<br>se<0.0001	p=0.22<br>R^2=9.06%<br>β=0.0138<br>CIs adj=<br>(-0.0134, 0.0409)<br>se=0.0111	p=0.49<br>R^2=0.00%<br>β= 0.0078<br>CIs adj=<br>(-0.0198, 0.0354)<br>se=0.0113	p=0.71<br>R^2=0.00%<br>β=0.002<br>CIs adj=<br>(-0.0102,<br>0.0142)<br>se=0.005	p=0.84<br>R^2=0.00[illegible]<br>β=-0.001[illegible]<br>CIs adj[illegible]<br>(-0.013[illegible]<br>0.0116[illegible]<br>se=0.00[illegible]
**Lack of regular exercise**	NA	NA	NA	NA	NA	NA

R^2: amount of heterogeneity accounted for; β: coefficient

ACHD, adults with congenital heart disease; CIs adj, Confidence intervals after adjustment; CHD, congenital heart disease; NA, not applicable

Cells in bold represent the statistically significant outcomes of these meta-analyses.

Supplementary Table S9. Assessment of the quality of evidence provided by the meta-analysis using the GRADE assessment tool

Questions:

1. Which is the prevalence of traditional risk factors for atherosclerotic cardiovascular disease in adults with congenital heart disease?

2. Is there difference in the risk factor prevalence between ACHD and controls?

**Outcome**	**Certainty assessment**							**Effect**	**Certainty**	**Importance**
	**No. of studies**	**Study design**	**Risk of bias**	**Inconsistency**	**Indirectness**	**Imprecision**	**Other considerations**	**Pooled prevalence (95% CI) & Risk ratio (95% CI)**		
**Hypertension prevalence**	34 (overall)<br>16 (with controls)	observational studies	not serious	very serious	serious	not serious	None	33% (26% to 40%) (overall)<br>RR 1.02 (0.92 to 1.12) (compared to controls)	⊕◯◯◯ Very Low	IMPORTANT
**Diabetes mellitus prevalence**	33 (overall)<br>17 (with controls)	observational studies	not serious	very serious	serious	serious	None	7% (5% to 9%) (overall)<br>RR 1.30 (1.09 to 1.55) (compared to controls)	⊕◯◯◯ Very Low	IMPORTANT
**Dyslipidemia prevalence**	27 (overall)<br>11 (with controls)	observational studies	not serious	very serious	serious	serious	None	17% (11% to 25%) (overall)<br>RR 0.99 (0.76 to 1.27) (compared to controls)	⊕◯◯◯ Very Low	IMPORTANT
**Obesity prevalence**	26 (overall)<br>12 (with controls)	observational studies	not serious	very serious	serious	not serious	None	18% (14% to 22%) (overall)<br>RR 1.01 (0.92 to 1.12) (compared to controls)	⊕◯◯◯ Very Low	IMPORTANT
**Smoking prevalence**	40 (overall)<br>18 (with controls)	observational studies	not serious	very serious	serious	not serious	strong association	12% (9% to 14%) (overall)<br>RR 0.67 (0.57 to 0.80) (compared to controls)	⊕◯◯◯ Very Low	IMPORTANT
**Physical inactivity prevalence**	18 (overall)<br>8 (with controls)	observational studies	not serious	very serious	serious	serious	None	54% (45% to 63%) (overall)<br>RR 1.16 (0.98 to 1.37) (compared to controls)	⊕◯◯◯ Very Low	IMPORTANT

95% CI: confidence interval; **RR:** Risk ratio

Supplementary Figures:

Supplementary Figure S1. Scatter plot of meta-regression analyses correlating hypertension (%) with age (years).

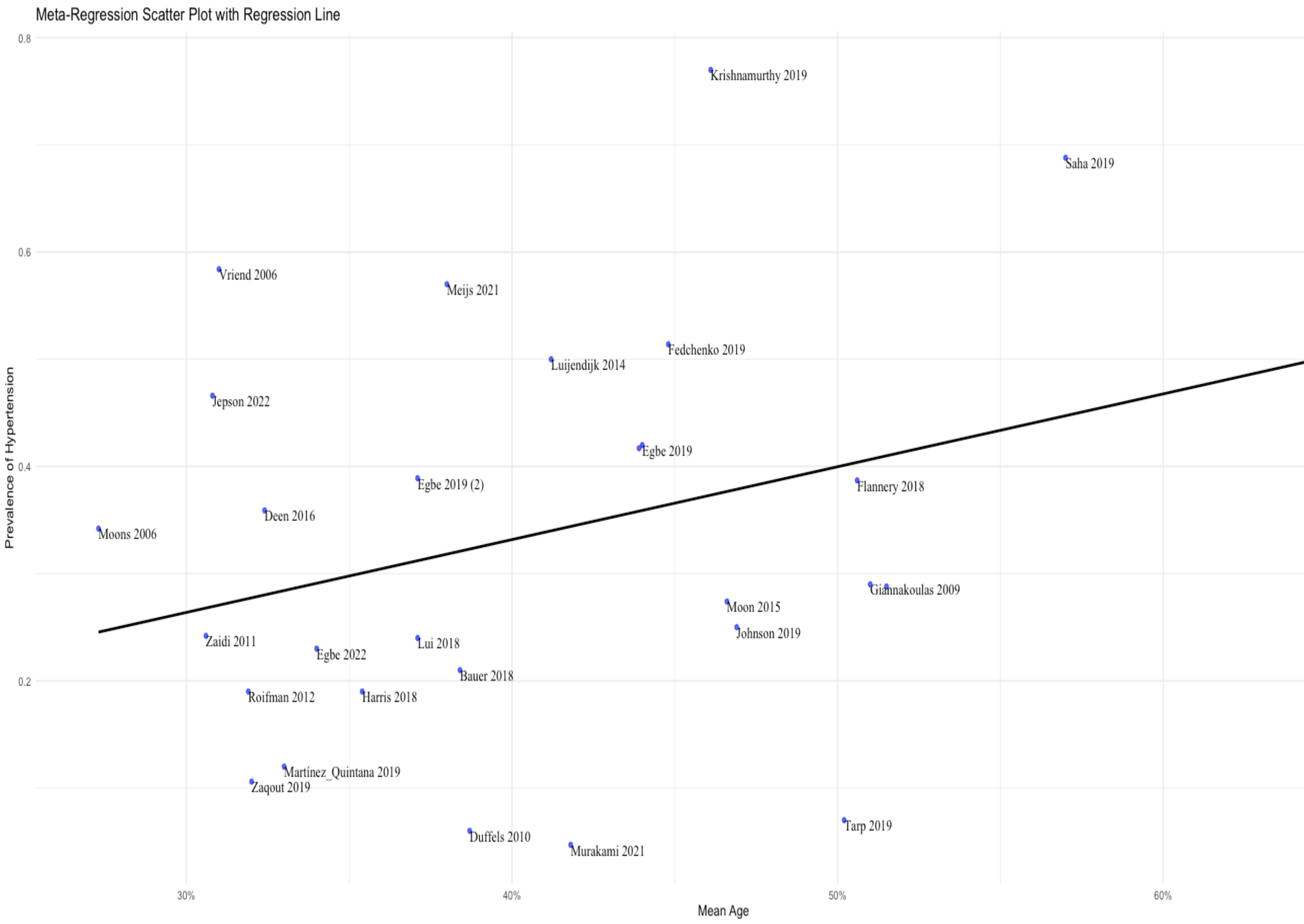

Supplementary Figure S2. Scatter plot of the meta-regression analysis correlating dyslipidemia (%) with age (years).

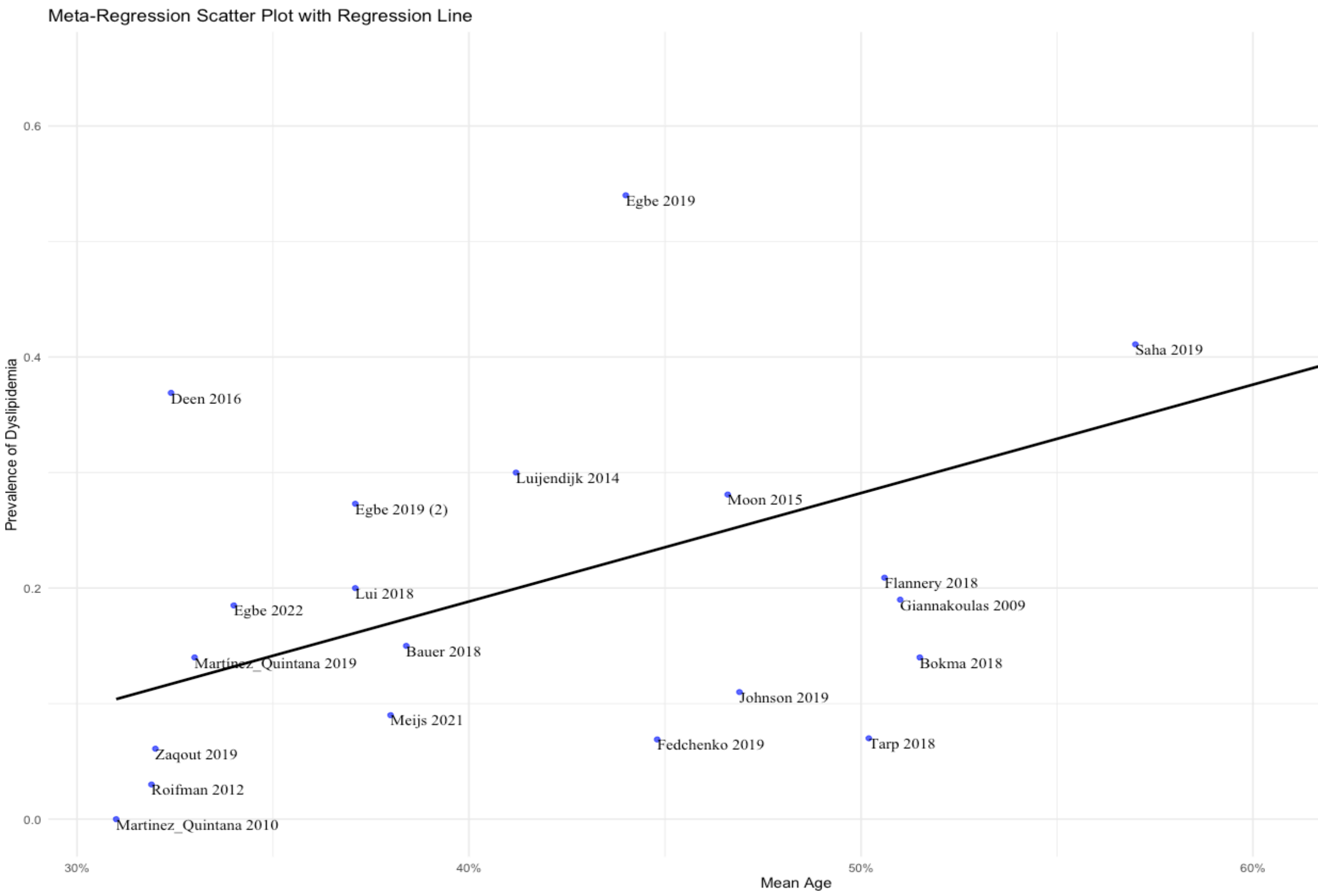

Supplementary Figure S3. Bubble plots of meta-regression analyses correlating dyslipidemia (%) with male gender (%).

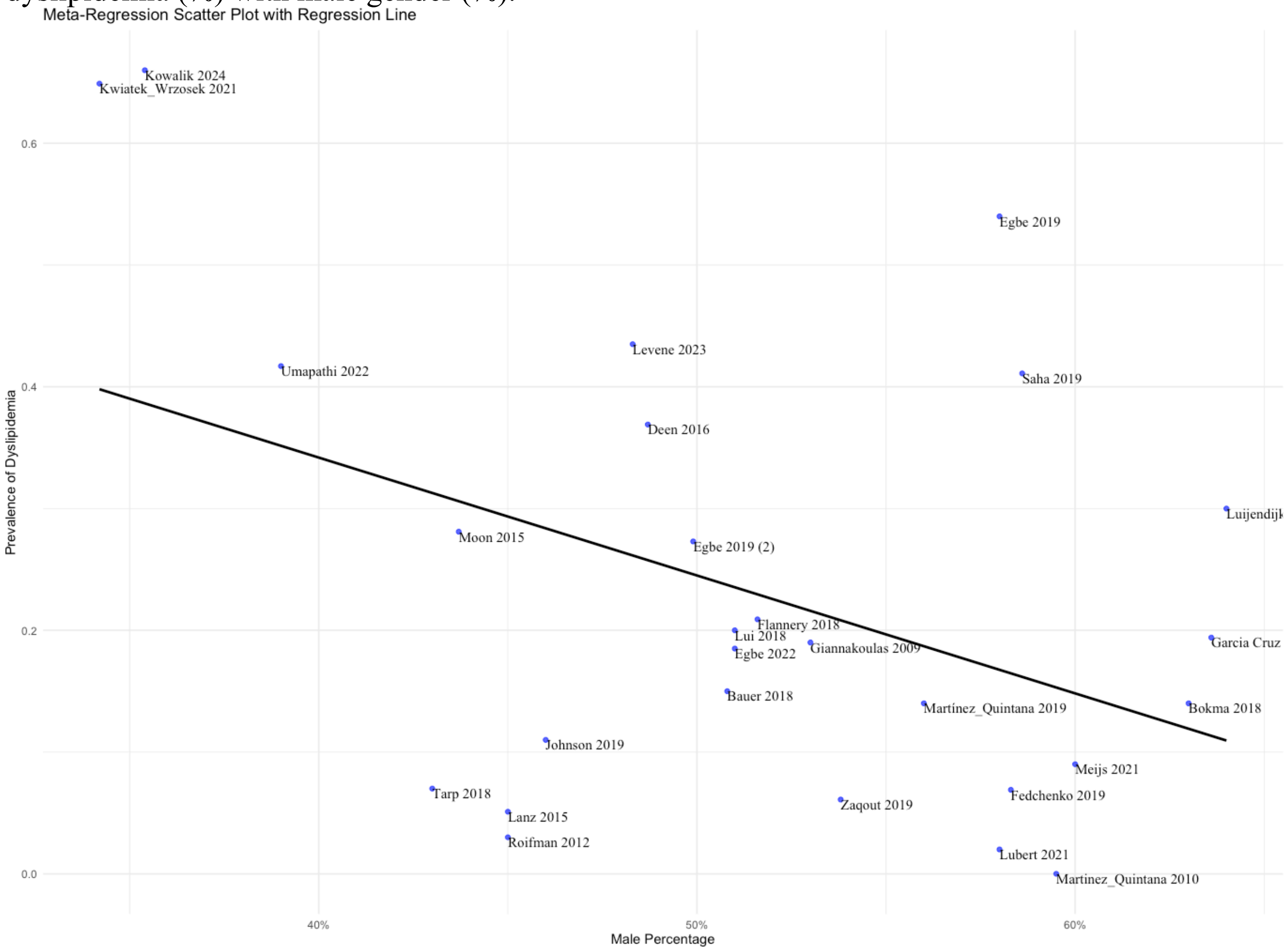

Supplementary Figure S4. Scatter plot of the meta-regression analysis correlating hypertension (%) with cyanotic disease (%).

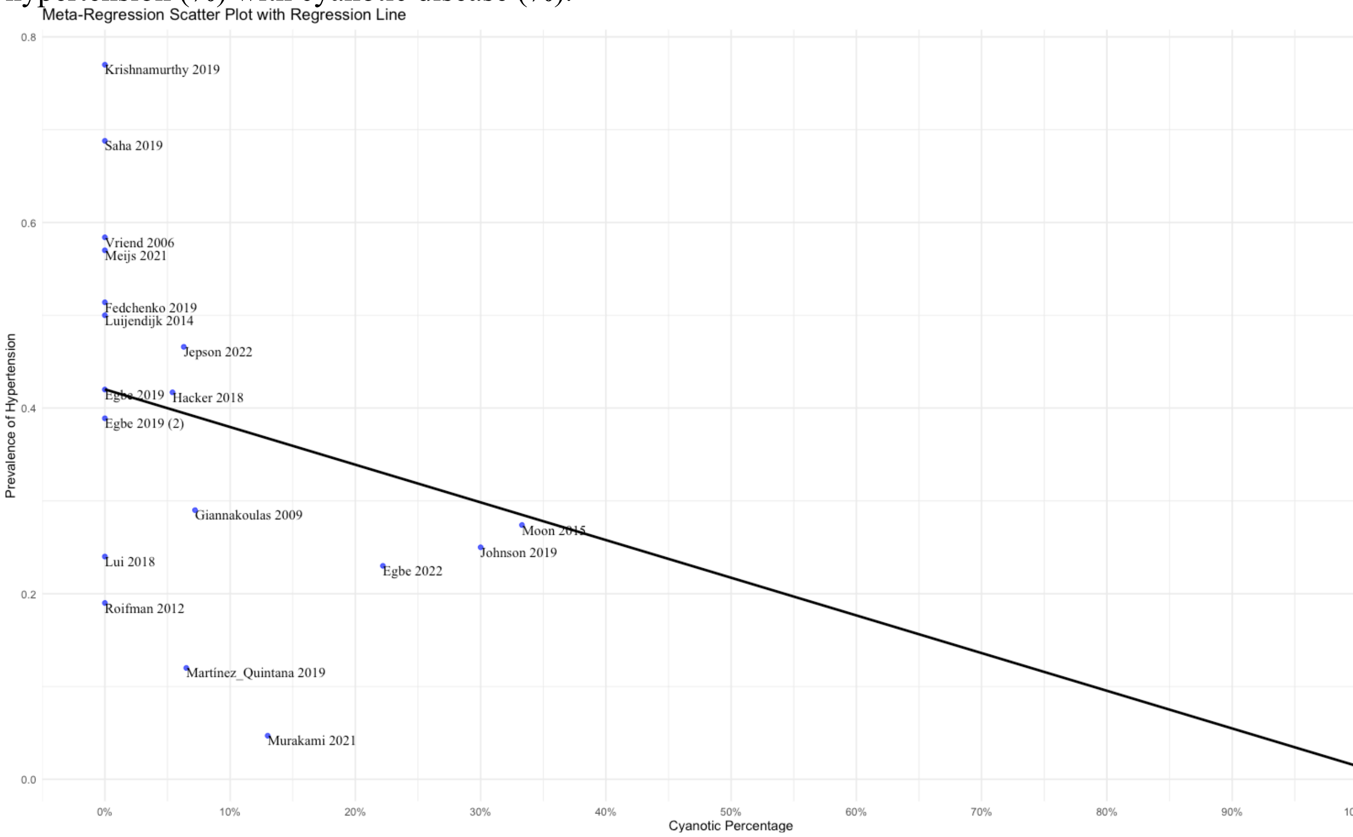

Supplementary Figure S5. Scatter plot of the meta-regression analysis correlating smoking (%) with great congenital heart disease complexity (%).

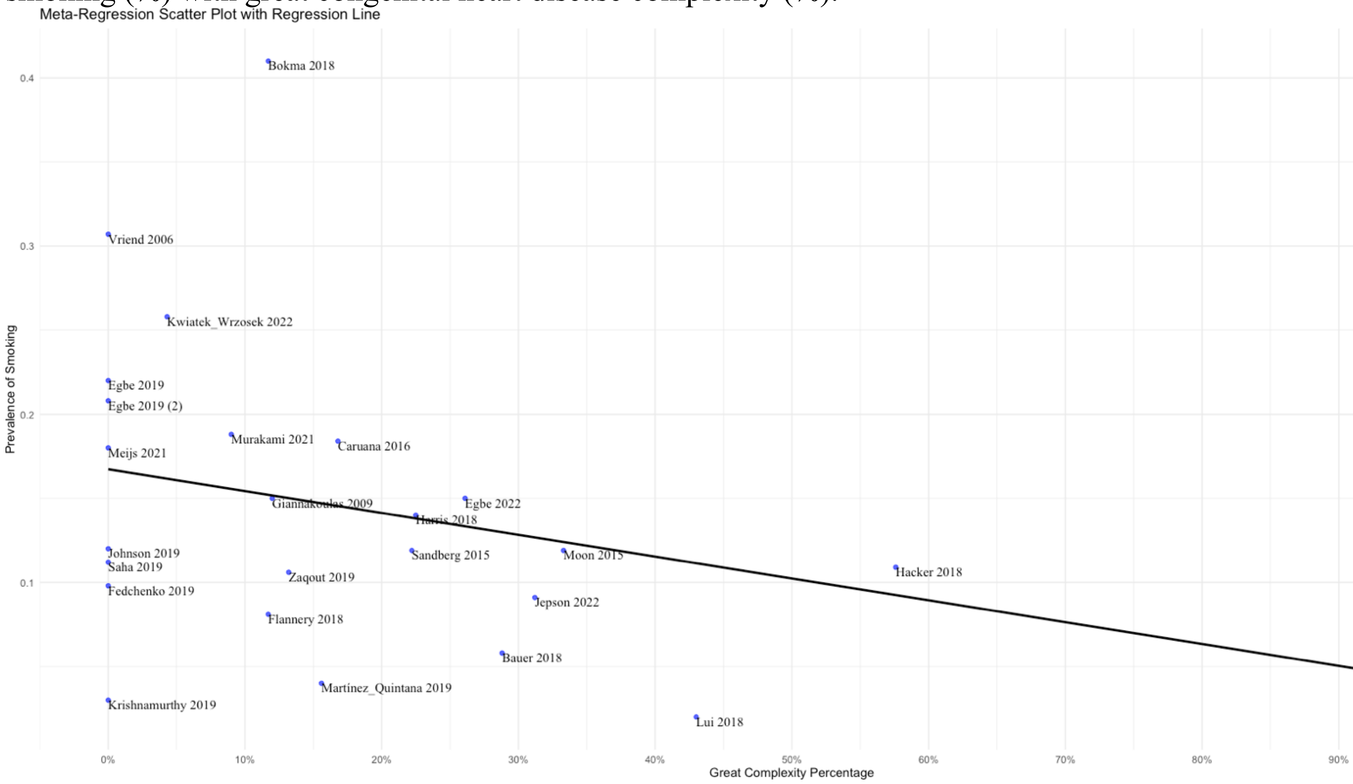

Supplementary Figure S6. Funnel plots of the meta-analysis of proportions for the assessment of atherosclerotic risk factor prevalence in adults with congenital heart disease.

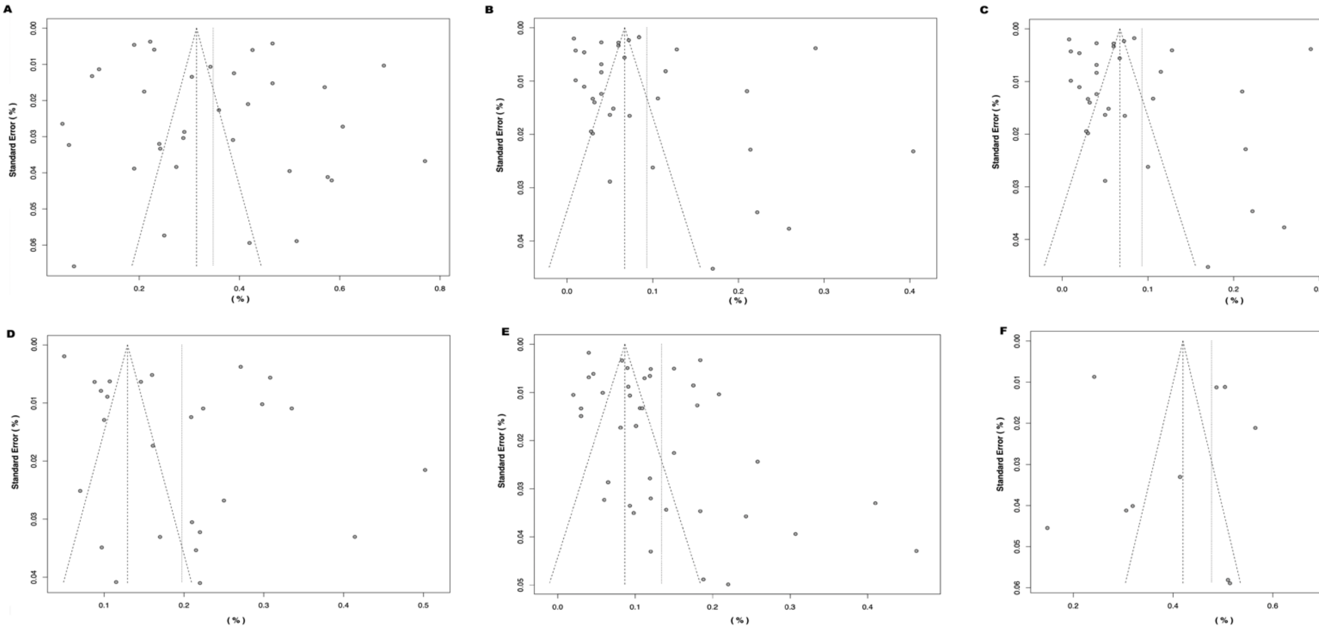

Supplementary Figure S7. Scatter plot of the meta-regression analysis correlating the risk ratio for hypertension with great congenital heart disease complexity (%).

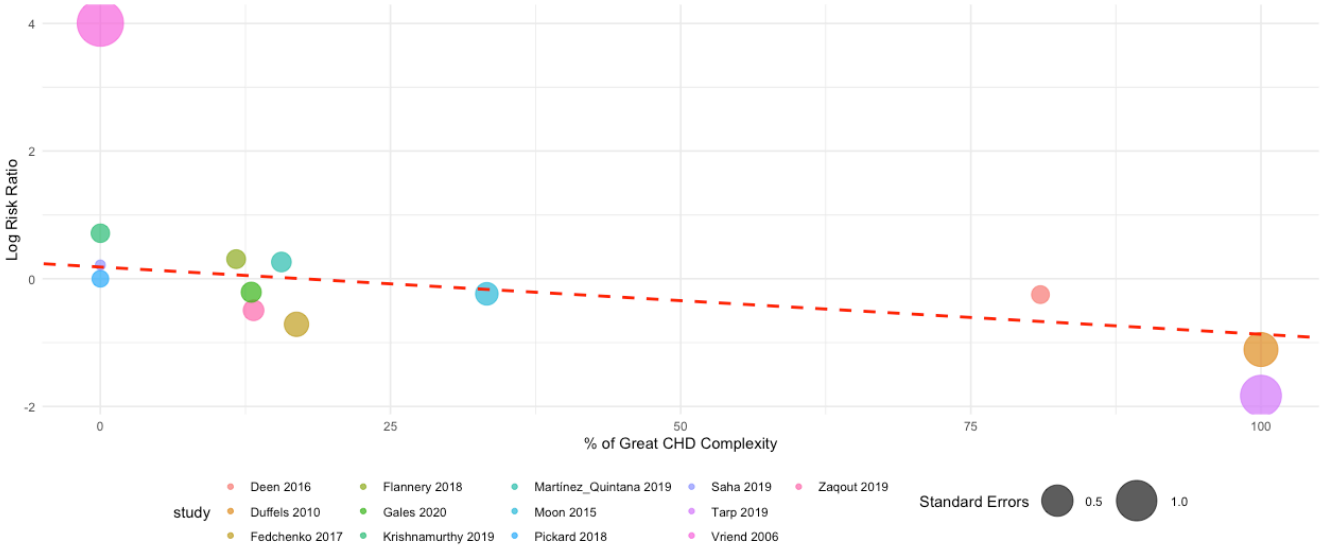

Supplementary Figure S8. Scatter plot of the meta-regression analysis correlating the risk ratio for diabetes mellitus with great congenital heart disease complexity (%).

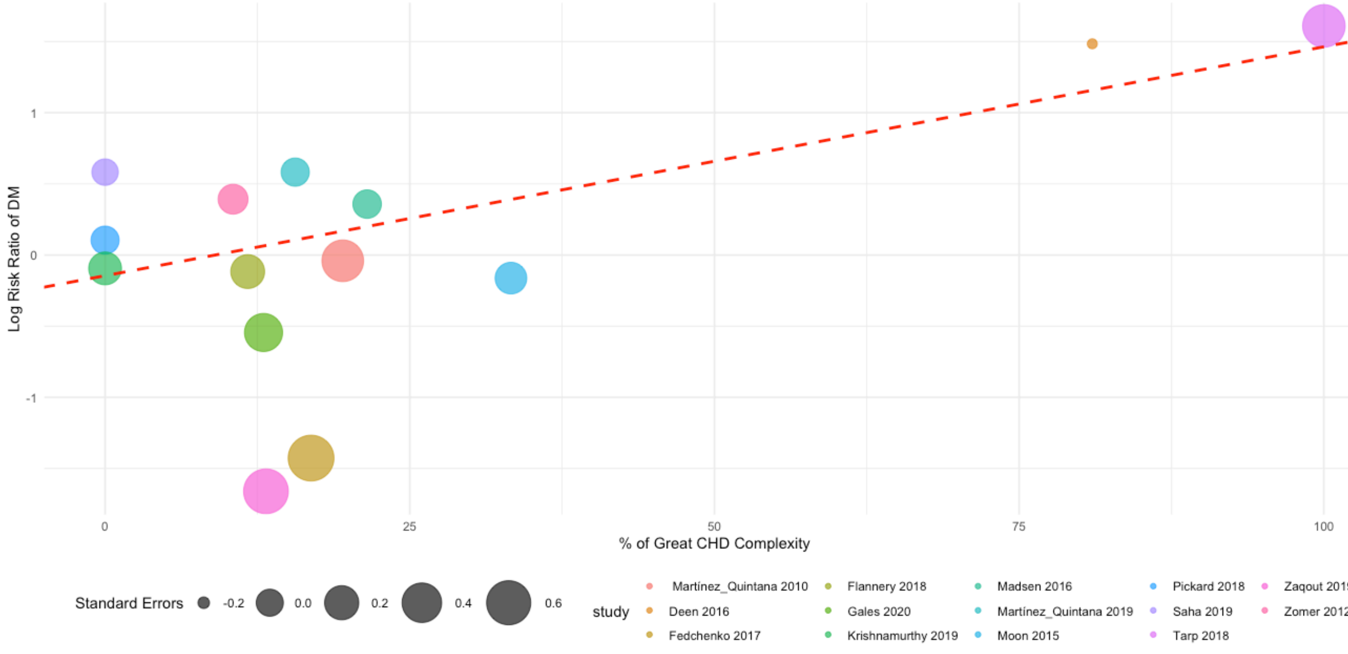

Supplementary Figure S9. Funnel plots of the meta-analysis of risk ratios for the comparison of atherosclerotic risk factor prevalence among adults with congenital heart disease and general population controls.

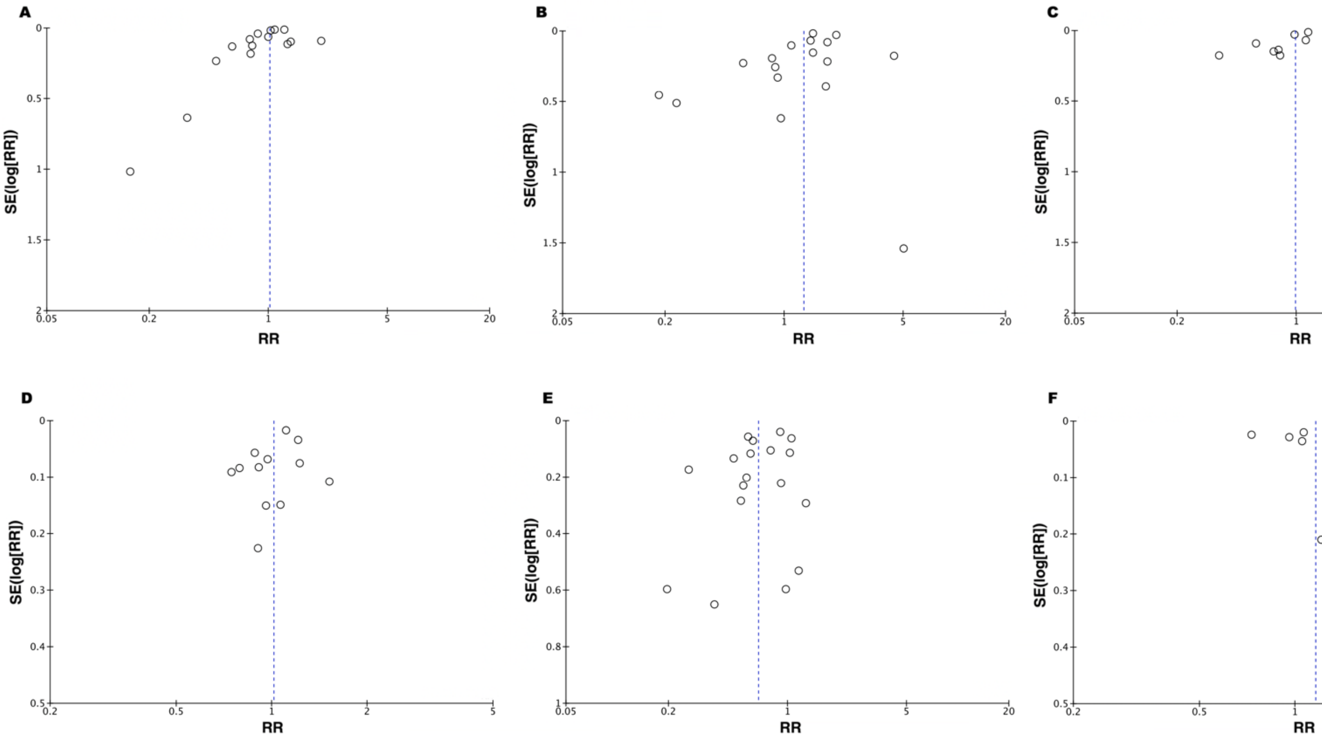

Supplement: Supplemental Appendix [file mmc1.pdf]
